# Alcohol Extracts From *Ganoderma lucidum* Delay the Progress of Alzheimer’s Disease by Regulating DNA Methylation in Rodents

**DOI:** 10.3389/fphar.2019.00272

**Published:** 2019-03-26

**Authors:** Guoxiao Lai, Yinrui Guo, Diling Chen, Xiaocui Tang, Ou Shuai, Tianqiao Yong, Dongdong Wang, Chun Xiao, Gailian Zhou, Yizhen Xie, Burton B. Yang, Qingping Wu

**Affiliations:** ^1^College of Pharmacy, Guangxi University of Chinese Medicine, Nanning, China; ^2^State Key Laboratory of Applied Microbiology Southern China – Guangdong Provincial Key Laboratory of Microbial Culture Collection and Application – Guangdong Open Laboratory of Applied Microbiology – Guangdong Institute of Microbiology, Guangzhou, China; ^3^Institute of Medical Science, University of Toronto, Toronto, ON, Canada

**Keywords:** DNA methylation, *Ganoderma lucidum*, aging, Alzheimer’s disease, active ingredients

## Abstract

Age-related changes in methylation are involved in the occurrence and development of tumors, autoimmune disease, and nervous system disorders, including Alzheimer’s disease (AD), in elderly individuals; hence, modulation of these methylation changes may be an effective strategy to delay the progression of AD pathology. In this study, the AD model rats were used to screen the main active extracts from the mushroom, *Ganoderma lucidum*, for anti-aging properties, and their effects on DNA methylation were evaluated. The results of evaluation of rats treated with 100 mg/kg/day of D-galactose to induce accelerated aging showed that alcohol extracts of *G. lucidum* contained the main active anti-aging extract. The effects on DNA methylation of these *G. lucidum* extracts were then evaluated using SAMP8 and APP/PS1 AD model mice by whole genome bisulfite sequencing, and some methylation regulators including Histone H3, DNMT3A, and DNMT3B in brain tissues were up-regulated after treatment with alcohol extracts from *G. lucidum*. Molecular docking analysis was carried out to screen for molecules regulated by specific components, including ganoderic acid Mk, ganoderic acid C6, and lucidone A, which may be active ingredients of *G. lucidum*, including the methylation regulators of Histone H3, MYT, DNMT3A, and DNMT3B. Auxiliary tests also demonstrated that *G. lucidum* alcohol extracts could improve learning and memory function, ameliorate neuronal apoptosis and brain atrophy, and down-regulate the expression of the AD intracellular marker, Aβ_1-42_. We concluded that alcohol extracts from *G. lucidum*, including ganoderic acid and lucidone A, are the main extracts involved in delaying AD progression.

## Introduction

DNA methylation by addition of a methyl group to cytosines followed by guanines [CpG sites] (5-methylcytosine [5mC]), is a mechanism of gene regulation. Methylation of CpG rich regions inhibits binding of gene regulatory machinery, leading to transcription silencing. Consequently, gene dysregulation via aberrant DNA methylation is an important contributor to disease. Evidence suggests that DNA methylation has an important role in neurological disorders, such as cognitive impairments (Sanchez-Mut et al., 2013), and alterations of DNA methylation contribute to the onset of Alzheimer’s disease (AD), since they were detected in autopsied brain DNA from 708 prospectively collected pre-symptomatic subjects (De Jager et al., 2014); global levels of 5mC and 5-hydroxymethylcytosine (5hmC; the first oxidative product in the demethylation of 5mC) were positively correlated with markers of AD, including amyloid beta, tau, and ubiquitin loads, and levels of 5mC and 5hmC were low in astrocytes and microglia, while they were elevated in neurons (Coppieters et al., 2014). While genomic DNA molecules are generally resistant to change in response to noncarcinogenic environmental factors, DNA methylation is more plastic and can be altered relatively easily by a variety of subtle exposures. Hence, regulation of such epigenetic changes is potentially an effective strategy to delay the progression of AD pathology.

*Ganoderma lucidum* is a Basidiomycetes fungus (order, Polyporales) that is popular as a medicinal mushroom and has been used since ancient times as a folk remedy in Asia, due to its diverse health-promoting properties (Cilerdžić et al., 2014; Cohen et al., 2014). *G. lucidum* is highly effective in decreasing cell senescence and has antioxidant properties (Deepalakshmi et al., 2013; Choi et al., 2014). The beneficial properties of *G. lucidum* are associated with a broad variety of bioactive compounds present in the fruiting body, mycelium, and spores, including polysaccharides, triterpenes, ganoderiols, sterols, amino acids, and nucleotides (Wang X.M. et al., 2014). In our previous study, we investigated *G. lucidum* metabolites and isolated 35 lanostane-type triterpenoids (Chen et al., 2017), including ganoderiols A, B, D, E, F, and M, and various ganoderic acids (β, A, B, C, C2, D2, d, E, F, G, J, H, and AM, among others) (Wang et al., 2010, 2017; Weng et al., 2011; Lee et al., 2016). APP/PS1 double transgenic mice express mutated human presenilin (DeltaE9) and human murine amyloid precursor protein (APPswe) fusions, both of which are present in familial AD patients. SAMP8 is a natural pathogenesis model associated with progressive aging, similar to AD. It is accompanied by the natural occurrence of dementia, brain atrophy, cortical and hippocampal neuronal shedding, and β-amyloid deposition. Both models are currently widely used for disease research in AD. In this study, to determine the pharmacologically active ingredients of *G. lucidum* and investigate their modes of action in AD and/or other age-related diseases, a rat model of aging rat was generated by intraperitoneal (i.p.) injection of 100 mg/kg/d D-galactose for 8 weeks (Zhong et al., 2016; Liang et al., 2017) and used to screen for the anti-aging effects of the main active extracts from *G. lucidum*. The effects of these extracts on DNA methylation were also evaluated by whole genome bisulfite sequencing of samples from SAMP8 and APP/PS1 mouse models of AD.

## Materials and Methods

### Preparation of *Ganoderma lucidum* Extracts

The fresh fruit bodies of *G. lucidum* were collected from the Mushroom Garden of Guangdong Yuewei Edible Fungi Technology Co., Ltd. and were identified by YX. A voucher specimen (No. GL20160322) has been deposited in the Herbarium of Microbiology Institute of Guangdong. The *Ganoderma lucidum* dried at 70°C and then ground into fine powders that could passed through a sieve with 30 pores per square inch.

5,000 g of powders of the *Ganoderma lucidum* were extracted with petroleum ether (1:10, w/v) in an incubator at 70°C for 1 h. Collected the extracted liquid and repeated the operation above once again, then combined extracted liquid. After cooling down to normal temperature, the extracted liquid were filtered, the filtrate was concentrated to dryness under vacuum using a rotary evaporator, then the *G. lucidum* oil extracts (OEG) were got.

**Figure 1:**
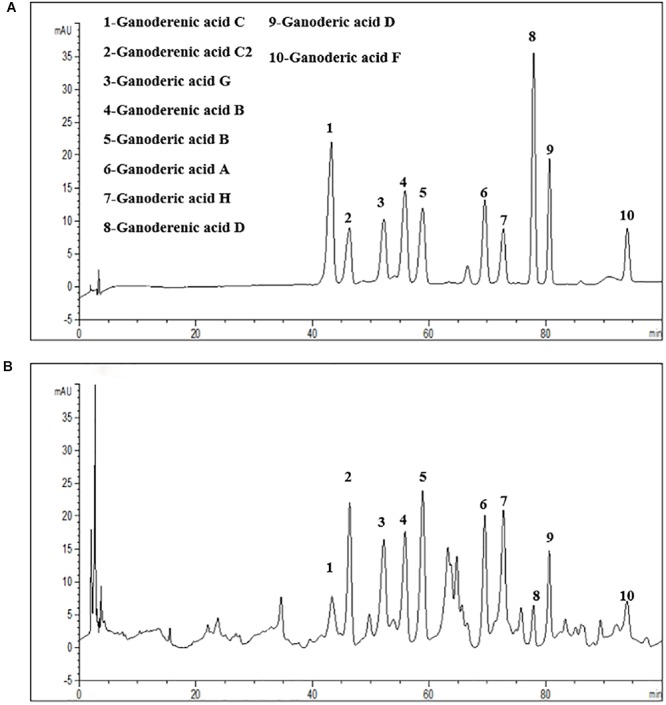
HPLC-UV analysis the *G. lucidum* lipophilic components. **(A)** Ten chemical reference substance from *G. lucidum*. **(B)** The HPLC-fingerprint of *G. lucidum* lipophilic components.

The filter residue were extracted with ethyl acetate in an incubator at 75°C for 2 h. Collected the extracted liquid and repeated the operation above once again, then combined extracted liquid. The filtrate was concentrated to dryness under vacuum using a rotary evaporator. Take part of the extract above to re-dissolve in methanol, and then added into a chromatographic column with absorbing material C18, use gradiently elution with water and methanol, collected the eluant of 40–75% aqueous methanol solution. Then the eluant was concentrated to dryness using a rotary evaporator under vacuum and then the extracts of *G. lucidum* small molecule compounds (AEG) were got, which stored at 4°C. The extracts of *G. lucidum* small molecule compounds (AEG) were analysis by HPLC-UV (Figure 1), mostly containing triterpenic acids and triterpene alcohols (Chen et al., 2017), ganoderenic acid C (0.177 mg/g), ganoderenic acid C2 (0.39 mg/g), ganoderic acid G (0.81 mg/g), ganoderenic acid B (0.30 mg/g), ganoderic acid B (0.75 mg/g), ganoderic acid A (1.46 mg/g), ganoderic acid H (2.24 mg/g), ganoderenic acid D (0.28 mg/g), ganoderic acid D (1.14 mg/g), ganoderic acid F (2.13 mg/g), then stored at 4°C.

The last filter residue were extracted with water in an incubator at 100°C for 2.5 h. Collected the extracted liquid and repeated the operation above once again, then combined extracted liquid. The filtrate was concentrated under vacuum using a rotary evaporator. Then the polysaccharides were precipitated from the filtrate in 80% aqueous ethanol (V/V) at 4°C overnight. The polysaccharides were centrifuged at 5,000 r/min for 10 min and further lyophilized in a freeze dryer (Alphai-4LD plus, CHRZST). The *G. lucidum* polysaccharides extracts (PEG) were got.

### Animal Model Generation and Treatment

Adult male Sprague-Dawley rats (180–220 g) obtained from the Center for Laboratory Animals, Guangdong Province (Certification No: SCXK [Yue] 2008-0020 and SYXK [Yue] 2008-0085), were pair-housed in plastic cages in a temperature-controlled room (25°C) with a 12/12-h light/dark cycle. Food and water were available *ad libitum*. All experimental protocols were approved by the Center for Laboratory Animals of the Guangdong Institute of Microbiology. All efforts were made to minimize the number of animals used.

For testing which main extracts of *G. lucidum* are active in anti-aging, a rat model of aging was generated by intraperitoneal (i.p.) injection of 100 mg/[kg⋅d] D-galactose for 8 weeks (Zhong et al., 2016; Liang et al., 2017) and treated with polysaccharide (PEG), alcohol (AEG), and oil (OEG) extracts from *G. lucidum* by gavage. Male rats were randomly divided into seven groups treated as follows: (1) control group, oral distilled water only; (2) model group, D-galactose (100 mg/[kg⋅d]); (3) low-dose AEG group (AEGL), D-galactose (100 mg/[kg⋅d]) + AEG (50 mg/[kg⋅d]); (4) high-dose AEG group (AEGH), D-galactose (100 mg/[kg⋅d]) + AEG (100 mg/[kg⋅d]); (5) low-dose PEG group (PEGL), D-galactose (100 mg/[kg⋅d]) + PEG (50 mg/[kg⋅d]); (6) high-dose PEG group (PEGH), D-galactose 100 mg/[kg⋅d] + PEG (100 mg/[kg⋅d]); (7) OEG group, D-galactose (100 mg/[kg⋅d]) + 0.5 ml OEG. Female rats were randomly divided into four groups treated as follows: (1) control group, oral distilled water; (2) model group, D-galactose (100 mg/[kg⋅d]) only; (3) low-dose AEG group, D-galactose (100 mg/[kg⋅d]) + AEG (50 mg/[kg⋅d]); (4) high-dose AEG group, D-galactose (100 mg/[kg⋅d]) + AEG (100 mg/[kg⋅d]). Rats were treated every day in the morning. Each group consisted of eight animals and treatment continued for 8 weeks.

Thirty male APP/PS1, 30 male SAMP8 transgenic mice (2 months old), and 10 C57BL/6J male mice with the same age and same genetic background were purchased from the Beijing HFK Bioscience Co. Ltd. [Certificate No: SCXK (Jing) 2014–0004]. The mean body weight of the mice was 20 ± 5 g. All animal experiments began at least 4 weeks after the animals arrived at our facility. Mice were randomly allocated into three groups: (1) AD model; (2) low-dose group, oral AEG (50 mg/[kg⋅d]); (3) high-dose group, intragastrically AEG (100 mg/[kg⋅d]) (*n* = 8 per group). The 10 C57BL/6J mice were used as the control group. The control and AD model groups were treated with volumes of distilled water equal to those of extracts (dissolved in distilled water) of *G. lucidum* used to treat mice. Treatments were administered intragastrically once daily for 24 weeks.

### Measurement of AD Parameters

The spatial learning and memory abilities of rats and mice were tested using a Morris water maze (MWM, DMS-2, Chinese Academy of Medical Sciences, Institute of Medicine). MWM tests included three periods, (1) initial spatial training, (2) spatial reversal training, and (3) the probe test, and were conducted as described previously (Chen et al., 2014). Animal weights were measured every 3 days during the drug administration period. Following water maze testing, blood and serum were acquired. Routine indices and cytokines (Wang et al., 2016) were measured and the brains of the animals dissected. Four brains from each group were fixed in 4% paraformaldehyde solution and prepared as paraffin sections.

Sections were stained with hematoxylin–eosin (HE) or by immunohistochemistry and observed by light microscopy (Zeng et al., 2013; Chen et al., 2014). Immunostaining for Aβ_1-42_, Histone H3, DNMT3A, and DNMT3B was performed using paraffin-embedded 4 μm sections with the two-step peroxidase conjugated polymer technique (DAKO Envision kit, DAKO, Carpinteria, CA, United States), and primary antibodies against Aβ_1-42_ (1:1000), Histone H3 (1:500), DNMT3A (1:1000), and DNMT3B (1:500).

### WGBS Library Preparation, Sequencing, Quality Analysis, and Mapping

Triplicate brain tissues samples were collected for each group and genomic DNA extracted using a Qiagen AllPrep Mini Kit, according to the manufacturer’s instructions. Isolated DNA samples were eluted in TE buffer, validated for quality and quantity using UV spectrophotometry, and stored long term at -80°C. DNA samples with an OD260/280 ratio between 1.75 and 1.85 were deemed high quality. Genomic DNA samples were sheared to 200–300 bp fragments using a Bioruptor Pico (Diagenode), with nine cycles of 30 s on and 30 s off. Suitable length DNA insertion fragments were prepared by interrupting 5 μg genomic DNA using high pressure nitrogen. DNA fragments with protruding terminals were repaired using 3′–5′ exonuclease and polymerase from the TruSeq Nano DNA LT Sample Prep Kit (Illumina). Next, an ‘A’ base was added to the 3′ end of repaired blunt DNA fragments, which were ligated to the labeled methylation adaptors. Conjugates were purified from agarose gels, and free and self-ligated molecules removed. Suitable DNA fragments were selected and treated with Bisulfite to convert unmethylated ‘C’ bases into ‘U’ bases. PCR was used to selectively enrich DNA fragments with adaptor molecules ligated to both ends, and the DNA library amplified. Products were quantified using the Agilent high sensitivity DNA assay on a Bioanalyzer 2100 system (Agilent). Libraries were then sequenced on the Hiseq platform (Illumina) by Shanghai Personal Biotechnology Corp. Ltd.

### Transcriptome Analysis

Total RNA was isolated using Trizol Reagent (Invitrogen Life Technologies) and the concentration, quality, and integrity determined using a NanoDrop spectrophotometer (Thermo Scientific). Three micrograms of RNA were used as input material for generation of sequencing libraries using the TruSeq RNA Sample Preparation Kit (Illumina, San Diego, CA, United States). Briefly, mRNA was purified from total RNA using poly-T oligo-attached magnetic beads. Fragmentation was carried out using divalent cations under elevated temperature in Illumina proprietary fragmentation buffer. First strand cDNA was synthesized using random oligonucleotides and SuperScript II. Subsequently, second strand cDNA synthesis was performed using DNA Polymerase I and samples treated with RNase H. Remaining overhangs were converted into blunt ends using exonuclease/polymerase enzymes, which were then removed. After adenylation of the 3′ ends of the DNA fragments, Illumina PE adapter oligonucleotides were ligated to prepare for hybridization. To select cDNA fragments of the preferred length (200 bp), libraries were purified using the AMPure XP system (Beckman Coulter, Beverly, CA, United States). DNA fragments with ligated adaptor molecules on both ends were selectively enriched using the Illumina PCR Primer Cocktail in a 15 cycle PCR reaction. Products were purified (AMPure XP system) and quantified using the Agilent high sensitivity DNA assay on a Bioanalyzer 2100 system (Agilent). Libraries were then sequenced on a Hiseq platform (Illumina) by Shanghai Personal Biotechnology Corp. Ltd.

Differentially expressed genes (DEGs) between treatment groups were identified by *de novo* assembly and annotation; expression levels of each transcript were measured according to the fragments/kb of exon per million mapped reads method. RSEM^1^ was used to quantify the abundance of genes and isoforms. The R statistical package software, EdgeR^2^, was used for analysis of differential expression. Functional enrichment analysis was performed to identify DEGs significantly enriched in Gene Ontology (GO) and metabolic pathways, using a Bonferroni-corrected *p*-value cut-off of ≤0.05, compared with the whole-transcriptome background. GO functional enrichment and Kyoto Encyclopedia of Genes and Genomes (KEGG) pathway analyses were performed using Goatools^3^ and KOBAS^4^, respectively (Wang et al., 2010; Cabili et al., 2011; Trapnell et al., 2013).

### Bisulfite Sequencing PCR (BSP)

In this study, two regions with relatively high levels of methylation in SAMP8, APP/PS1, and Control mice, were randomly chosen to validate the results of WGBS. Primers used to amplify *AKT2* (Chromosome 1: 84411726–84450162+) and *MAPK3* (Chromosome 1: 15412603–15613746+) are illustrated in Figure 5. Bisulfite conversion of genomic DNA was performed using an EpiTect^®^ Plus DNA Bisulfite Kit (59124, Qiagen). The PCR program was as follows: initial denaturation at 94°C for 5 min; 30 cycles of 94°C for 30 s, 56°C annealing for 30 s, and 72°C for 30 s; and a final extension at 72°C for 5 min. PCR products were separated and detected on 1% agarose gel, purified using a QIA quick Gel Extraction Kit (28704, Qiagen), cloned into linearized vector using T_4_ DNA Ligase, and transformed into *Trans*-T1 cells. Ten subclones were selected for each fragment and subsequently sequenced using the ABI 3730XL sequencing platform.

### Statistical Analysis

All data are described as the means ± standard deviations (SD) of at least three independent experiments. The significance of differences between treatments were analyzed by one-way analysis of variance (ANOVA) tests, with *p* < 0.05 considered significant, using statistical package for the social sciences (SPSS, Abacus Concepts, Berkeley, CA, United States) and Prism5 (GraphPad, San Diego, CA, United States) software.

## Results

### Screening the Main *G. lucidum* Active Extracts for Anti-aging Properties

**Figure 2:**
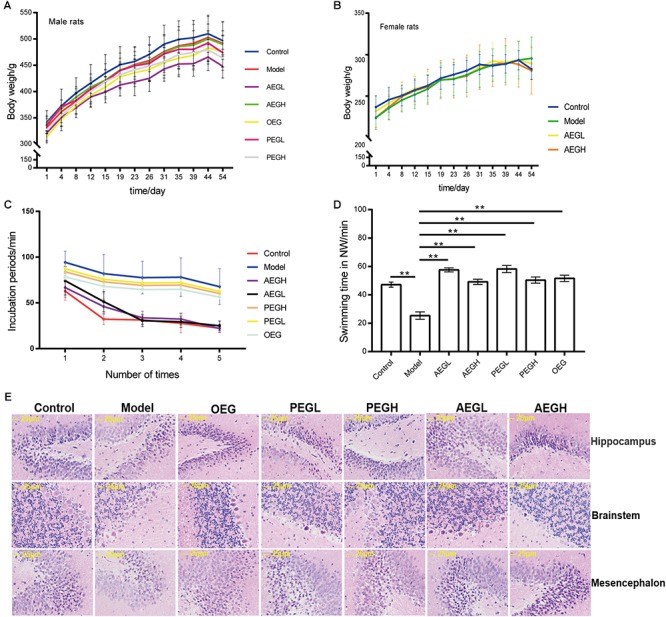
Screening the main active extracts from *Ganoderma lucidum* extracts on anti-aging in the D-galactose induced deficient rats. The male rats were randomly divided into seven groups as follows: (1) control group, oral distilled water only; (2) model group, D-galactose (100 mg/[kg⋅d]); (3) low-dose AEG group (AEGL), D-galactose (100 mg/[kg⋅d]) + AEG (50 mg/[kg⋅d]); (4) high-dose AEG group (AEGH), D-galactose (100 mg/[kg⋅d]) + AEG (100 mg/[kg⋅d]); (5) low-dose PEG group (PEGL), D-galactose (100 mg/[kg⋅d]) + PEG (50 mg/[kg⋅d]); (6) high-dose PEG group (PEGH), D-galactose 100 mg/[kg⋅d] + PEG (100 mg/[kg⋅d]); (7) OEG group, D-galactose (100 mg/[kg⋅d]) + 0.5 ml OEG. Female rats were randomly divided into four groups treated as follows: (1) control group, oral distilled water; (2) model group, D-galactose (100 mg/[kg⋅d]) only; (3) low-dose AEG group, D-galactose (100 mg/[kg⋅d]) + AEG (50 mg/[kg⋅d]); (4) high-dose AEG group, D-galactose (100 mg/[kg⋅d]) + AEG (100 mg/[kg⋅d]), polysaccharide (PEG), alcohol (AEG), and oil (OEG) extracts from *G. lucidum* by gavage. Every group consisted of eight animals and the procedure duration was 8 weeks. **(A)** Male animals weighed; **(B)** While female rats weighed; **(C,D)** Morris water maze tests; **(E)** Brain tissue HE staining. ^∗∗^*p* < 0.01.

The fur of animals treated with *G. lucidum* extracts was much smoother than that of those in the model group. The average weight of animals did not differ significantly between the treated and model groups (*p* > 0.05); male animals weighed approximately 320 g at the beginning and 500 g at the end of the experiment (Figure 2A), while female rats weighed approximately 240 g at the beginning and 290 g at the end of the experiment (Figure 2B).

In MWM tests, compared to the model group (100.11 ± 9.37 s), the incubation periods for each of the groups treated with *G. lucidum* extracts treated were longer or shorter. On the first day, the incubation period for the low-dose AEG group was 86.37 ± 11.46 s and that for the high-dose group was 82.00 ± 19.44 s, which were significantly shorter than the model group (*p* < 0.01). On the fourth day, the incubation period for the low-dose AEG group was 39.30 ± 5.63 s and that for the high-dose group was 30.74 ± 3.69 s, and the differences were significant relative to the model group (*p* < 0.01; Figure 2C), demonstrating that administration of AEG could ameliorate D-galactose-induced learning and memory dysfunction in rats and that the main active anti-aging extract from *G. lucidum* resides in the alcohol extracts.

The swimming time of the control group in the NW quadrant (47.20 ± 1.82 s) was significantly longer than that in the other three quadrants (*p* < 0.01). The swimming time in the NW quadrant of the model group was 25.23 ± 2.66 s, which was significantly shorter than that of the control group (*p* < 0.01), suggesting that the rats remembered the location of the placement of the platform. The swimming durations of the low- and high-dose AEG groups were 57.55 ± 1.39 s and 49.16 ± 1.87 s, respectively, which were significantly longer than the model group. Compared with the model group, the differences were significant (*p* < 0.01; Figure 2D). The other extracts treated groups do not improved so much (Figure 2).

In the control group, pyramidal cells in the CA1 region were arranged precisely and tightly, and no cell loss was observed. Additionally, in the control group, the cells were round and intact with clearly stained, dark blue nuclei (Figure 2E). In contrast, in the model groups, noticeable damage in the hippocampus was observed by histopathology. The layered pyramidal structure was disrupted, and neuronal loss was observed in the CA1 region, and there were neurons with pyknotic nuclei and shrunken or irregular shape. These abnormalities were reduced by treatment with AEG. There were greater numbers of cells with superior cell morphology in the AEG-treated group compared with the untreated groups; with a greater difference in the AEGH group compared with the AEGL group. Together, these results demonstrate that the main active anti-aging extract from *G. lucidum* resides in the alcohol extracts.

### WGBS of AEG-Treated D-Galactose-Induced Deficient Rats

**Figure 3:**
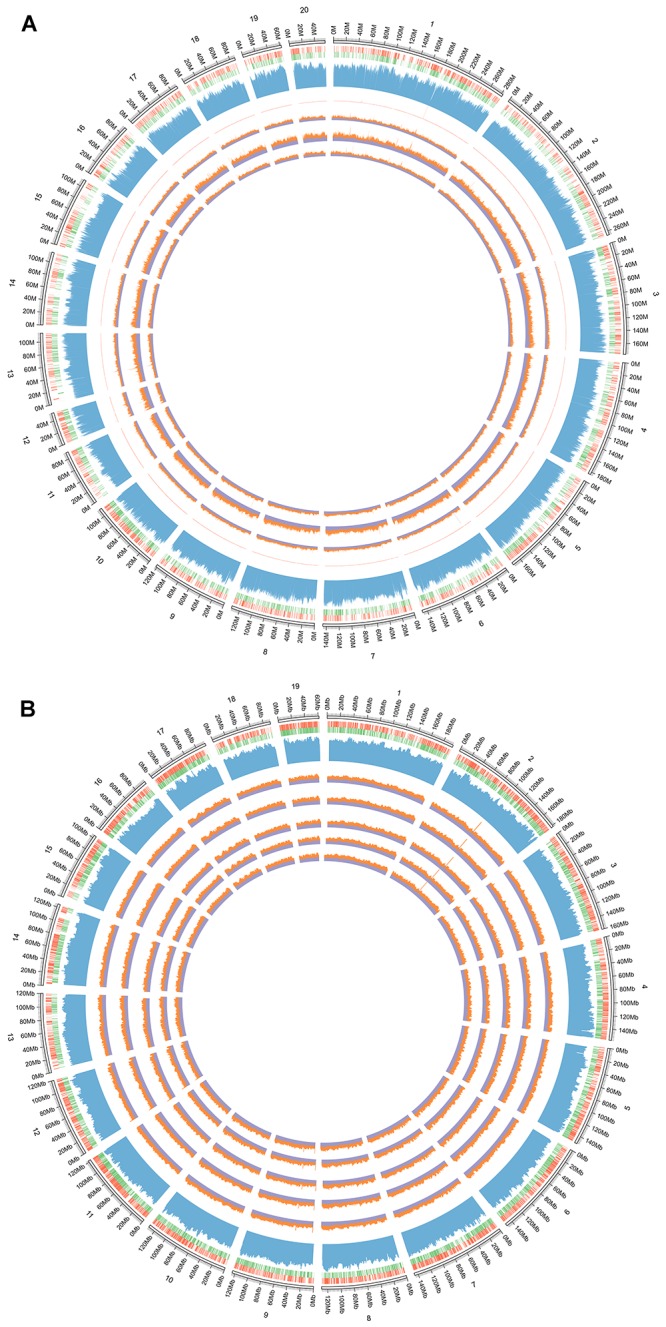
**(A)** The whole genome methylation circular map of D-galactose induced deficient rats. From the outside to the inner: the first circle is genome; the second circle is the distribution of genome, the red circle is positive chain gene and the green is negative chain gene; the third circle is the distribution of genomic GC content; the fourth is the sequencing depth of all samples; the fifth circle is the control group, the sixth is the AEG-treated group, and the seventh is the model group. Control group that received oral distilled water, model group that received intraperitoneal injection (i.p.) of 100 mg/[kg⋅d] D-galactose, AEG-treated group that received D-galactose (100 mg/[kg⋅d]) + AEG (100 mg/[kg⋅d]). **(B)** The whole genome methylation circular map of APP/PS1 and SAMP8 mice. From the outside to the inner: the first circle is genome of mice; the second circle is the distribution of genome, the red circle is positive chain gene and the green is negative chain gene; the third circle is the distribution of genomic GC content; the fourth is the SAMP8 model group, the fifth is AEG-treated group on SAMP8 mice, the sixth is the AEG-treated group on APP/PS1 mice, and the seventh is the APP/PS1 model group, the eighth is the control group. Control and model group that received oral distilled water; AEG-treated group at a dosage of 100 mg/[kg⋅d] in AEG, and the extracts were dissolved in distilled water and were given by intragastric administration once daily for 24 weeks, polysaccharide (PEG), alcohol (AEG), and oil (OEG) extracts from *G. lucidum* by gavage.

Three whole brains dissected from each of the D-galactose-induced model, AEG-treated, and control rats were pooled to provide one sample per group and analyzed by WGBS. As shown in Table 1, there were fewer methylated CpG sites in the model group (74.9%) than in controls (77.5%), whereas the AEG-treated group recovered to a value of 76.5%, indicating that excessive D-galactose inhibits DNA methylation changes in the brain and that alcohol extracts from *G. lucidum* can ameliorate this dysregulation (*p* < 0.05). Details of the methylated strands and the distributions of methylated genes are presented in Tables 2, 3, respectively. A whole genome methylation circular map generated using Circos (Krzywinski et al., 2009) is provided in Figure 3A.

**Table 1:** Cytosine context methylation analysis of brain tissues in AEG-treated D-galactose-induced deficient rats.

Sample	Total cytosine	Type	CpG	CHG	CHH	Unknown context
AEG	15,534,176,963	Methylated C	595,389,651	149,008,458	479,858,609	7,652
		Unmethylated C	183,141,321	3,462,346,734	10,664,432,190	115,883
		percentage	76.5%	4.1%	4.3%	6.2%
Model	17,010,810,890	Methylated C	641,046,353	164,343,883	535,114,480	9,011
		Unmethylated C	214,607,050	3,724,896,568	11,730,802,556	130,840
		percentage	74.9%	4.2%	4.4%	6.4%
Control	14,965,478,059	Methylated C	598,105,218	151,106,866	475,851,826	7,641
		Unmethylated C	174,027,353	3,400,594,438	10,165,792,358	108,386
		percentage	77.5%	4.3%	4.5%	6.6%

*Alcohol (AEG) extracts from *G. lucidum* by gavage*.

**Table 2:** Methylated strand analysis of brain tissues in AEG-treated D-galactose-induced deficient rats.

Sample	Type	Counts	Description
AEG	CT/GA/CT	133,585,197	[(Converted) top strand]
	GA/CT/CT	0	[Complementary to (converted) top strand]
	GA/CT/GA	0	[Complementary to (converted) bottom strand]
	CT/GA/GA	131,851,325	[(Converted) bottom strand]
Model	CT/GA/CT	144,205,567	[(Converted) top strand]
	GA/CT/CT	0	[Complementary to (converted) top strand]
	GA/CT/GA	0	[Complementary to (converted) bottom strand]
	CT/GA/GA	142,325,935	[(Converted) bottom strand]
Control	CT/GA/CT	126,675,501	[(Converted) top strand]
	GA/CT/CT	0	[Complementary to (converted) top strand]
	GA/CT/GA	0	[Complementary to (converted) bottom strand]
	CT/GA/GA	124,160,178	[(Converted) bottom strand]

*Alcohol (AEG) extracts from *G. lucidum* by gavage*.

**Table 3:** The distribution of methylated genes analysis of brain tissues in AEG-treated D-galactose-induced deficient rats.

Sample	Group	Total_bases	Tag_count	Tags/Kb
Control	CDS_Exons	39,977,515	8,921,362	223.16
	5’UTR_Exons	3,002,257	602,843	200.8
	3’UTR_Exons	13,709,583	2,927,376	213.53
	Introns	817,405,745	163,673,356	200.24
	TSS_up_1kb	27,119,038	3,934,857	145.1
	TSS_up_5kb	123,811,475	18,469,306	149.17
	TSS_up_10kb	226,283,650	33,787,938	149.32
	TES_down_1kb	27,042,568	4,078,199	150.81
	TES_down_5kb	118,479,240	17,748,831	149.81
	TES_down_10kb	212,554,470	31,527,752	148.33
AEG	CDS_Exons	39,977,515	11,261,049	281.68
	5’UTR_Exons	3,002,257	562,354	187.31
	3’UTR_Exons	13,709,583	2,853,633	208.15
	Introns	817,405,745	167,349,858	204.73
	TSS_up_1kb	27,119,038	4,018,422	148.18
	TSS_up_5kb	123,811,475	19,016,199	153.59
	TSS_up_10kb	226,283,650	34,838,221	153.96
	TES_down_1kb	27,042,568	4,120,303	152.36
	TES_down_5kb	118,479,240	18,083,259	152.63
	TES_down_10kb	212,554,470	32,215,190	151.56
Model	CDS_Exons	39,977,515	12,861,620	321.72
	5’UTR_Exons	3,002,257	710,562	236.68
	3’UTR_Exons	13,709,583	3,171,679	231.35
	Introns	817,405,745	182,489,824	223.25
	TSS_up_1kb	27,119,038	4,522,338	166.76
	TSS_up_5kb	123,811,475	20,747,720	167.58
	TSS_up_10kb	226,283,650	37,789,603	167
	TES_down_1kb	27,042,568	4,517,278	167.04
	TES_down_5kb	118,479,240	19,633,194	165.71
	TES_down_10kb	212,554,470	34,863,583	164.02

*Alcohol (AEG) extracts from *G. lucidum* by gavage*.

Bioinformatic analysis of GO and KEGG enrichment for genes with methylation affected by extracts of *G. lucidum* demonstrated that it can influence the estrogen signaling pathway, steroid hormone biosynthesis, long-term depression, renin secretion, the cGMP-PKG signaling pathway, glutamatergic synapses, B cell receptor signaling, serotonergic synapses, prolactin signaling, insulin secretion, gap junctions, GnRH signaling, phospholipase D signaling, cholinergic synapses, TNF signaling, retinol metabolism, long-term potentiation, purine metabolism, thyroid hormone synthesis, and sphingolipid signaling. Additional details are shown in Supplementary Tables S1–S3. These WGBS results demonstrate that alcohol extracts from *G. lucidum* can regulate DNA methylation in the brains of D-galactose-induced deficient rats.

### Effects of AEG on DNA Methylation inAPP/PS1 and SAMP8 Mice

To further understand the mechanism of action of AEG on DNA methylation, the whole brains dissected from SAMP8, AEG-treated SAMP8, APP/PS1, AEG-treated APP/PS1 and control mice, three per group, were pooled to generate one sample per group, and analyzed by WGBS. As shown in Table 4, the percentage of methylated CpG sites in the SAMP8 group was 73.7% and that in APP/PS1 mice was 76.0%, which were both lower than the control value (77.5%), while the AEG-treated groups showed recovery to 74.7 and 77.1%, indicating that alcohol extracts from *G. lucidum* can ameliorate dysregulation of methylation, particularly in APP/PS1 mice, where the effects were significant (*p* < 0.05). Details of the methylated strands and the distributions of methylated genes are presented in Tables 5, 6, respectively. A circular whole genome methylation map generated using Circos is presented in Figure 3B.

**Table 4:** Cytosine context methylation of AEG on DNA methylation in APP/PS1 and SAMP8 mice.

Sample	Total cytosine	Type	CpG	CHG	CHH	Unknown context
SAMP8	12,072,778,86	Methylated C	281,115,405	100,751,628	330,787,246	20
		Unmethylated C	100,512,967	1,906,796,619	6,245,659,817	364
		Percentage	73.7%	5.0%	5.0%	5.2%
AEG-SAMP8	15,534,176,963	Methylated C	271,926,316	96,803,506	318,028,942	18
		Unmethylated C	91,944,767	1,845,639,001	6,064,557,392	345
		Percentage	74.7%	5.0%	5.0%	5.0%
AEG-APP/PS1	17,010,810,890	Methylated C	287,541,533	95,762,475	314,480,144	19
		Unmethylated C	85,633,466	1,875,234,074	6,152,398,180	374
		Percentage	77.1%	4.9%	4.9%	4.8%
APP/PS1	12,072,778,866	Methylated C	308,957,749	101,474,938	330,703,258	27
		Unmethylated C	97,761,611	2,004,916,312	6,502,006,323	395
		Percentage	76.0%	4.8%	4.8%	6.4%
Control	14,965,478,059	Methylated C	598,105,218	151,106,866	475,851,826	7,641
		Unmethylated C	174,027,353	3,400,594,438	10,165,792,358	108,386
		Percentage	77.5%	4.3%	4.5%	6.6%

*Alcohol (AEG) extracts from *G. lucidum* by gavage*.

**Table 5:** Methylated strand of AEG on DNA methylation in APP/PS1 and SAMP8 mice.

Sample	Type	Counts	Description
SAMP8	CT/GA/CT	77,675,742	[(Converted) top strand]
	GA/CT/CT	0	[Complementary to (converted) top strand]
	GA/CT/GA	0	[Complementary to (converted) bottom strand]
	CT/GA/GA	75,938,750	[(Converted) bottom strand]
AEG-SAMP8	CT/GA/CT	75,970,545	[(Converted) top strand]
	GA/CT/CT	0	[Complementary to (converted) top strand]
	GA/CT/GA	0	[Complementary to (converted) bottom strand]
	CT/GA/GA	74,617,468	[(Converted) bottom strand]
AEG-APP/PS1	CT/GA/CT	77,022,081	[(Converted) top strand]
	GA/CT/CT	0	[Complementary to (converted) top strand]
	GA/CT/GA	0	[Complementary to (converted) bottom strand]
	CT/GA/GA	75,298,340	[(Converted) bottom strand]
APP/PS1	CT/GA/CT	81,608,876	[(Converted) top strand]
	GA/CT/CT	0	[Complementary to (converted) top strand]
	GA/CT/GA	0	[Complementary to (converted) bottom strand]
	CT/GA/GA	79,632,061	[(Converted) bottom strand]
Control	CT/GA/CT	126,675,501	[(Converted) top strand]
	GA/CT/CT	0	[Complementary to (converted) top strand]
	GA/CT/GA	0	[Complementary to (converted) bottom strand]
	CT/GA/GA	124,160,178	[(Converted) bottom strand]

*Alcohol (AEG) extracts from *G. lucidum* by gavage*.

**Table 6:** The distribution of methylated genes of AEG on DNA methylation in APP/PS1 and SAMP8 mice.

Sample	Group	Total_bases	Tag_count	Tags/Kb
SAMP8	CDS_Exons	92,770,739	10,520,478	113
	5’UTR_Exons	6,817,714	675,919	99
	3’UTR_Exons	30,830,041	3,147,020	102
	Introns	1,469,823,225	154,457,401	105
	TSS_up_1kb	29,802,851	1,894,436	64
	TSS_up_5kb	133,470,634	8,597,890	64
	TSS_up_10kb	239,135,684	15,695,506	66
	TES_down_1kb	31,715,036	1,963,976	62
	TES_down_5kb	137,780,250	8,829,933	64
	TES_down_10kb	242,815,081	16,089,066	66
AEG-SAMP8	CDS_Exons	92,770,739	10,262,220	110.62
	5’UTR_Exons	6,817,714	662,568	97.18
	3’UTR_Exons	30,830,041	3,082,650	99.99
	Introns	1,469,823,225	150,628,394	102.48
	TSS_up_1kb	29,802,851	1,858,553	62.36
	TSS_up_5kb	133,470,634	8,429,922	63.16
	TSS_up_10kb	239,135,684	15,383,988	64.33
	TES_down_1kb	31,715,036	1,929,853	60.85
	TES_down_5kb	137,780,250	8,680,528	63
	TES_down_10kb	242,815,081	15,790,406	65.03
AEG-APP/PS1	CDS_Exons	92,770,739	9,966,285	107.43
	5’UTR_Exons	6,817,714	650,467	95.41
	3’UTR_Exons	30,830,041	3,040,614	98.63
	Introns	1,469,823,225	149,238,024	101.53
	TSS_up_1kb	29,802,851	1,829,107	61.37
	TSS_up_5kb	133,470,634	8,285,235	62.08
	TSS_up_10kb	239,135,684	15,097,669	63.13
	TES_down_1kb	31,715,036	1,896,834	59.81
	TES_down_5kb	137,780,250	8,520,685	61.84
	TES_down_10kb	242,815,081	15,519,964	63.92
APP/PS1	CDS_Exons	92,770,739	11,036,360	118.96
	5’UTR_Exons	6,817,714	687,146	100.79
	3’UTR_Exons	30,830,041	3,215,666	104.3
	Introns	1,469,823,225	158,021,162	107.51
	TSS_up_1kb	29,802,851	1,926,517	64.64
	TSS_up_5kb	133,470,634	8,726,063	65.38
	TSS_up_10kb	239,135,684	15,913,380	66.55
	TES_down_1kb	31,715,036	2,005,028	63.22
	TES_down_5kb	137,780,250	8,981,573	65.19
	TES_down_10kb	242,815,081	16,369,504	67.42
Control	CDS_Exons	92,770,739	9,678,453	104.33
	5’UTR_Exons	6,817,714	640,382	93.93
	3’UTR_Exons	30,830,041	2,988,843	96.95
	Introns	1,469,823,225	146,269,536	99.52
	TSS_up_1kb	29,802,851	1,798,551	60.35
	TSS_up_5kb	133,470,634	8,120,448	60.84
	TSS_up_10kb	239,135,684	14,814,071	61.95
	TES_down_1kb	31,715,036	1,863,605	58.76
	TES_down_5kb	137,780,250	8,358,614	60.67
	TES_down_10kb	242,815,081	15,226,423	62.71

*Alcohol (AEG) extracts from *G. lucidum* by gavage*.

The results of bioinformatic analysis of GO and KEGG enrichment of genes with differential methylation in treated mice are shown in Supplementary Tables S2–S4. These data show that *G. lucidum* can influence the adipocytokine signaling pathway, adrenergic signaling in cardiomyocytes, aldosterone synthesis and secretion, circadian entrainment, dopaminergic synapses, glutamatergic synapses, the GnRH signaling pathway, pancreatic secretion, the phosphatidylinositol signaling system, serotonergic synapses, and the thyroid hormone signaling pathway. More details are presented in Supplementary Tables S5, S6. Overall, these results show that alcohol extracts from *G. lucidum* can regulate DNA methylation in the brains of APP/PS1 and SAMP8 AD model mice.

### mRNA Sequencing and Analysis

**Figure 4:**
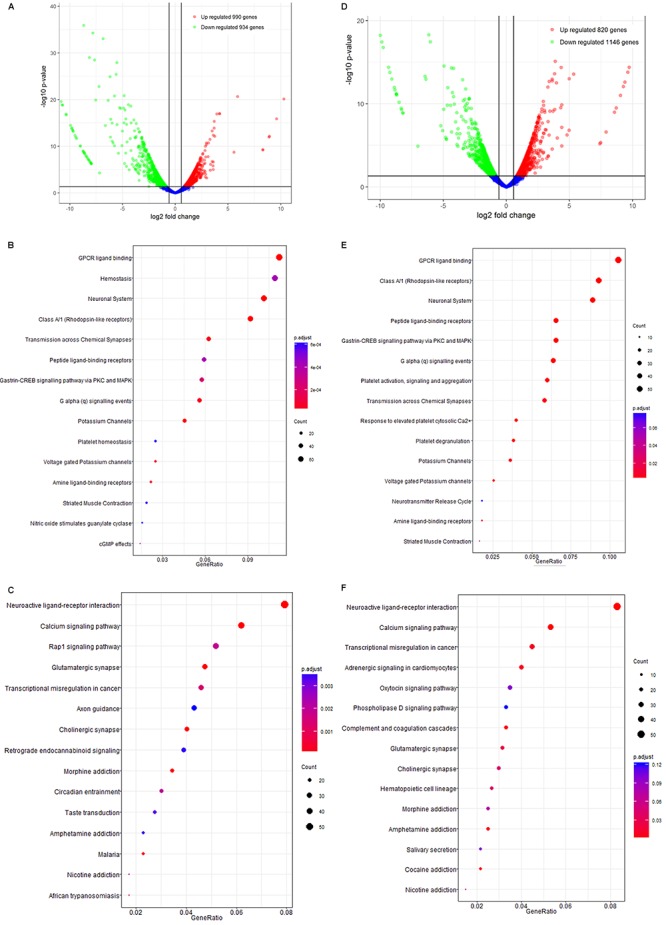
The mRNA analysis of APP/PS1 and SAMP8 mice brain tissues. **(A–C)** is for APP/PS1; **(D–F)** is for SAMP8. Control and model group that received oral distilled water, AEG-treated group at a dosage of 100 mg/[kg⋅d] in AEG, and the extracts were dissolved in distilled water and were given by intragastric administration once daily for 24 weeks, alcohol (AEG) extracts from *G. lucidum* by gavage.

In APP/PS1 mice, 14732 mRNAs were identified as expressed in the brain, among which 1924 exhibited differential expression between the control and model groups (fold-change > 1.50, *p* < 0.05, Supplementary Table S4). Of these 1,924 DEGs, 990 were up-regulated and 934 were down-regulated (Figure 4A and Supplementary Table S7). Bioinformatic analysis of GO and KEGG enrichment indicated involvement in GPCR ligand binding, class A/1 (rhodopsin-like receptors), the neuronal system, peptide ligand-binding receptors, neuroactive ligand-receptor interaction, calcium signaling, Rap 1 signaling, and glutamatergic synapses (Figure 4B,C).

Similarly, 14723 mRNAs were identified in the brains of SAMP8 mice, among which 1966 were differentially expressed in controls vs. the model group (fold-change > 1.50, *p* < 0.05, Supplementary Table S4). Of these, 820 were up-regulated and 1146 down-regulated (Figure 4D). The results of bioinformatic analysis of GO and KEGG enrichment are shown in Figure 4E,F and indicate enrichment for GPCR ligand binding, hemostasis, the neuronal system, class A/1 (rhodopsin-like receptors), neuroactive ligand-receptor interaction, calcium signaling, transcriptional misregulation in cancer, and adrenergic signaling in cardiomyocytes.

Overall, these data indicate that the alcohol extracts from *G. lucidum* can influence AD progression via multiple targets and certainly warrant further study.

### BSP Confirmation

**Figure 5:**
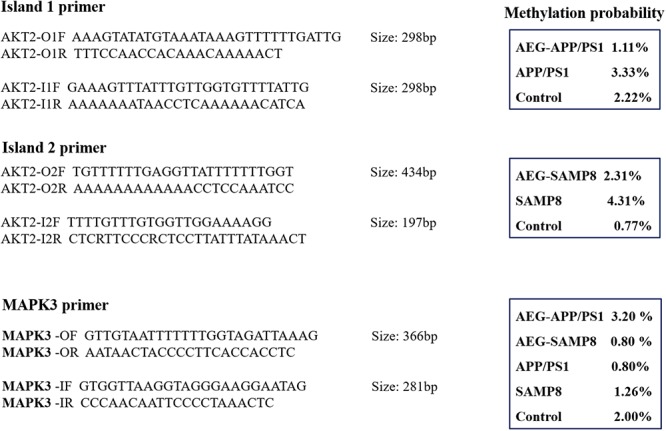
Bisulfite sequencing PCR results of AKT2 (Chromosome 1: 84411726-84450162+) and MAPK3 (Chromosome 1: 15412603-15613746+) in APP/PS1 and SAMP8 mice brain tissues. Control and model group that received oral distilled water, AEG-treated group at a dosage of 100 mg/[kg⋅d] in AEG, and the extracts were dissolved in distilled water and were given by intragastric administration once daily for 24 weeks, alcohol (AEG) extracts from *G. lucidum* by gavage.

We used BSP to detect the methylation of CpG at the *AKT2* and *MAPK3* loci and compared the results for *G. lucidum*-treated and control mice (Figure 5). The methylation of *AKT2* island 1 was 1.11% in AEG-APP/PS1 mice, 3.33% in APP/PS1 mice, and 2.22% in controls (*p* < 0.05, Figure 5). *AKT2* island 2 was 2.31% in AEG-SAMP8, 4.31% in SAMP8, and 0.77% in control mice (*p* < 0.05, Figure 5). Moreover, methylation of *MAPK3* was 3.20% in AEG-APP/PS1, 0.80% in AEG-SAMP8, 0.80% in APP/PS1, 1.26% in SAMP8, and 2.00% in control mice. Taken together, these data indicate a high level of consistency between the BSP and WGBS analyses and demonstrate that alcohol extracts of *G. lucidum* can regulate DNA methylation.

### Validation of the Negative Regulation of DNA Methyltransferases

**Figure 6:**
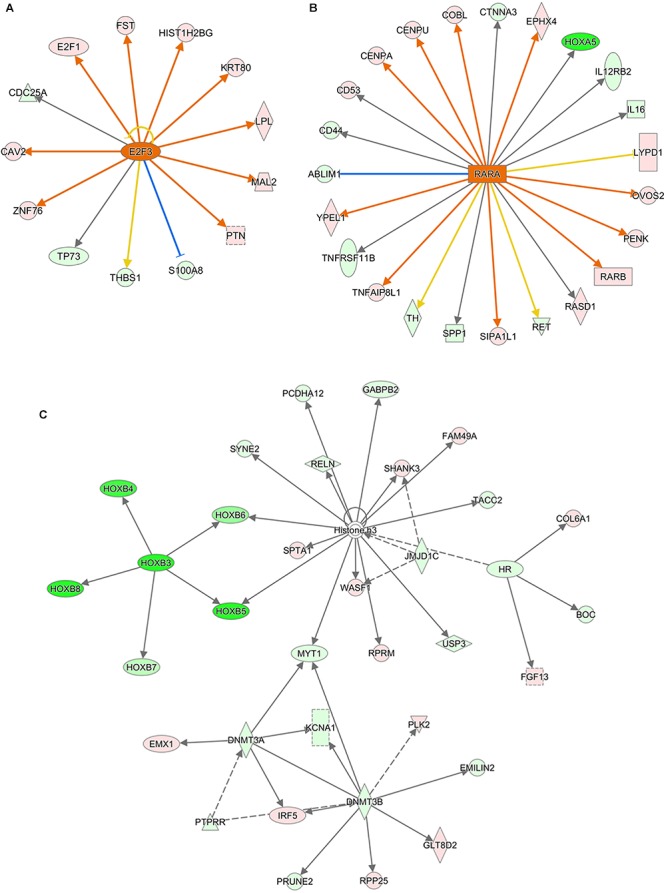
The networks of Ingenuity Canonical Pathways results of mRNA of APP/PS1 and SAMP8 mice brain tissues. Control and model group that received oral distilled water, AEG-treated group at a dosage of 100 mg/[kg⋅d] in AEG, and the extracts were dissolved in distilled water and were given by intragastric administration once daily for 24 weeks, alcohol (AEG) extracts from *G. lucidum* by gavage. **(A)** is the increased E2F3 signaling_upstream_regulator by AEG; **(B)** is increased RARA signaling_upstream_regulator by AEG and **(C)** is the Network of different expression mRNA using IPA pathway analysis.

**Figure 7:**
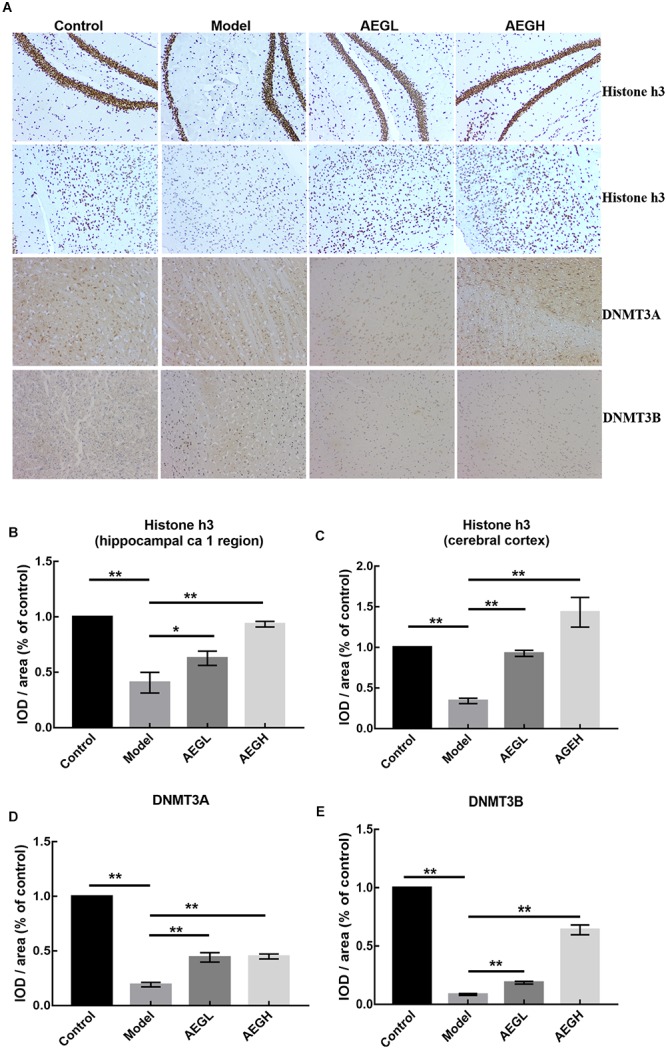
The expression of Histone H3, DNMT3A and DNMT3B protein in the brain of alcohol extracts from *G. lucidum* treated APP/PS1 mice detected using IHC. Control and model group that received oral distilled water. Dosage of 50 mg/[kg⋅d] is AEGL, and 100 mg/[kg⋅d] is AEGH, and the extracts were dissolved in distilled water and were given by intragastric administration once daily for 24 weeks, alcohol (AEG) extracts from *G. lucidum* by gavage. **(A)** Brain tissue IHC detection; **(B–E)** Average optical density of brain tissue IHC detection. ^∗^*p* < 0.05 and ^∗∗^*p* < 0.01.

Ingenuity Canonical Pathways and Networks analysis of mRNA results for AEG-APP/PS1, APP/PS1, and control group mice showed that molecules in the dataset targeted by the upstream regulator *Akt* included *BCL2A1*, *BMF*, *BTG2*, *CD44*, *CDH1*, *COL3A1*, *FOS*, *KCNA5*, *LCN2*, *MCAM*, *MMP9*, *PPP1R1B*, *RAG1*, and *RASD1*; a MAPK12-targeted molecule in the dataset was *MMP9*; HOXB3 targeted molecules in dataset included *HOXB4*, *HOXB5*, *HOXB6*, *HOXB7*, and *HOXB8* (Figure 6); DNMT3A-targeted molecules were *CDH1*, *CNR1*, *EMX1*, *IRF5*, *KCNA1*, and *MYT1* (Figure 6); DNMT3B-targeted molecules were *CDH1*, *CNR1*, *EMILIN2*, *FBXL16*, *GLT8D2*, *IRF5*, *KCNA1*, *KDELR3*, *MYT1*, *PRKCB*, *PRUNE2*, and *RPP25* (Figure 6); and P38 MAPK-targeted molecules in the dataset included *ANK1*, *CD44*, *CD86*, *COL3A1*, *CRHR1*, *DIO2*, *FAS*, *FOS*, *FST*, *KDELR3*, *LBX1*, *MMP9*, *PPM1D*, *RUNX2*, and *TOP2A*. We measured the expression of Histone H3 (A, B for CA1 area and C for motor cortex), DNMT3A (A, C), and DNMT3B (A, D) in brain tissues of APP/PS1 and SAMP8 mice using immunohistochemistry, and the results showed that these DNA methyltransferases were up-regulated after treatment with alcohol extracts from *G. lucidum* (Figure 7), indicating that *G. lucidum* can influence DNA methylation levels and that this may be an important signaling pathway influenced by *G. lucidum* in mediating its effects in delaying AD progression and/or aging.

### Measurement of AD Related Markers

**Figure 8:**
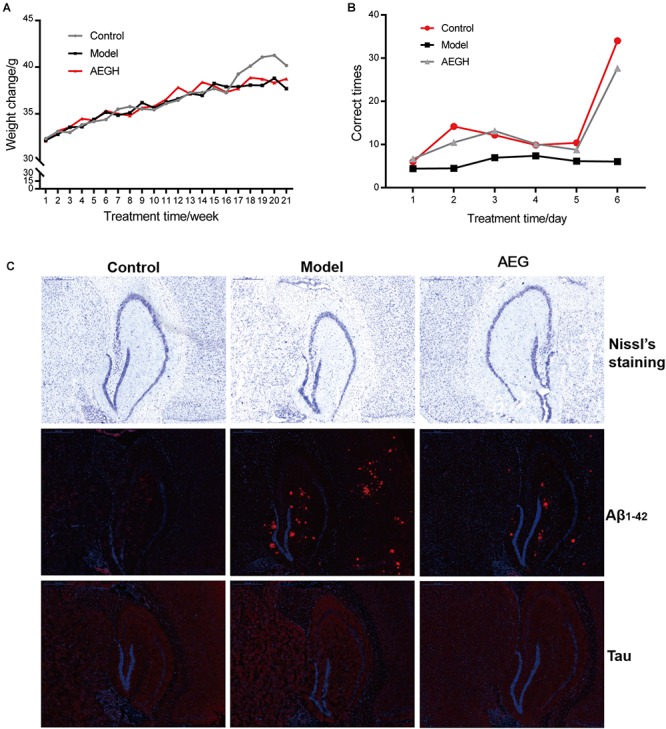
Effects of alcohol extracts from *Ganoderma lucidum* on APP/PS1 mice. **(A)** Body weight changes. **(B)** Escape corrected times analysis in the H type maze. **(C)** Nissl staining, expression of Aβ_1-42_ and Tau using immunofluorescence methods. AEG-treated group at a dosage of 100 mg/[kg⋅d] in AEG, and the extracts were dissolved in distilled water and were given by intragastric administration once daily for 24 weeks, alcohol (AEG) extracts from *G. lucidum* by gavage.

Animals weighed approximately 32 g at the beginning and 38 g at the end of the experiment (Figure 8A). Behavioral tests showed that alcohol extracts from *G. lucidum* (AEG-treated group) could significantly ameliorate learning and memory abilities, resulting in much improved times relative to the model group (Figure 8B, *p* < 0.05). Histopathological examination showed that treatment with alcohol extracts from *G. lucidum* could reduce the swelling of brain tissue, as Nissl’s staining indicated that the CA1 area was larger, and there was an increased number of neurons (Figure 8C, *p* < 0.05 vs. model). Immunohistochemistry analysis indicated that alcohol extracts from *G. lucidum* could down-regulate the expression of the intracellular marker of AD, Aβ_1-42_, as there were fewer red fluorescent foci in brain tissues (Figure 8C, *p* < 0.05 vs. model).

## Discussion

Although analysis of methylation cannot reveal all the complex mechanisms underlying aging, it can reveal important information about the regulation of key genes related to aging and/or age-related diseases. Hence, exploration of methylation may be an effective approach to understanding the true causes of aging, through investigation of the molecular mechanisms involved in age-related methylation changes. Since the mechanisms underlying aging are not yet fully understood, such information is potentially of great clinical relevance and the delay of aging and/or prevention of age-related disease through determining optimal methylation states in aging, via measurement of whole genome hypomethylation and hypermethylation of specific genes, will be an important future research direction (Harrison and Dexter, 2013). In this context, we have explored natural small molecule compounds, and screened those with potential effects on the regulation of DNA methylation. *G. lucidum*, a folk remedy in Asia, exhibits marked effects in decreasing cell senescence and has antioxidant properties. In this study, we found that alcohol extracts from *G. lucidum* can improve D-galactose-induced learning and memory dysfunction in rats and ameliorate neuron apoptosis and brain atrophy. WGBS analysis showed that methylation levels of CpG sites in model group animals were 74.9%, which was lower than that of controls (77.5%), while in the AEG-treated group, levels recovered to 76.5%. Our experiments also demonstrated that *G. lucidum* alcohol extracts could improve learning and memory abilities, ameliorate neuron apoptosis and brain atrophy, and down-regulate the expression of the AD intracellular marker, Aβ_1-42_ in APP/PS1 AD model mice, while positive effects on DNA methylation were observed in SAMP8 and APP/PS1 mice.

Recent studies suggest that DNA methylation, a major epigenetic mechanism, is pivotal in the pathogenesis of age-related neurodegeneration and cognitive disorders (Konsoula and Barile, 2012). Moreover, epigenome alterations in adult somatic tissue may reflect age-associated deleterious events. Accumulation of genomic damage can lead to chromosomal instability and telomere shortening, reactive oxygen species-induced damage to mitochondrial functions and consequent reduced energy production, stem cell depletion, accumulation of damaged proteins via loss of proteostasis, processes leading to senescence and changes in intercellular communications and, finally, epigenome alterations. Many of these altered pathways are considered primary factors in age-related diseases, including cancer, neurodegenerative diseases, atherosclerosis, and inflammation. AD is associated with dysregulation of DNA methylation, histone modifications, and non-coding RNAs (De Jager et al., 2014; Zusso et al., 2018). Further, recent findings point to a role for 5hmC in the development of diseases, including AD, since global levels of 5mC and 5hmC are positively correlated with one another and with markers of AD, including amyloid beta, tau, and ubiquitin loads, potentially opening new pathways for AD treatment through correction of methylation and hydroxymethylation alterations (Coppieters et al., 2014). Hypermethylation targets include thromboxane A2 receptor (*TBXA2R*), sorbin, SH3 domain containing 3 (*SORBS3*), and spectrin beta 4 (*SPTBN4*), indicating that the cyclic AMP response element-binding protein (CREB) activation pathway and the axon initial segment may contribute to AD pathology (Sanchez-Mut et al., 2013). Akt2 ablation protects against cardiac aging through restoration of Foxo1-related autophagy and mitochondrial integrity (Ren et al., 2017). Moreover, DNA methylation is one possible epigenetic variation correlated with the occurrence of neural-tube defects, and AKT2 is a candidate gene associated with neural-tube defects (Ma et al., 2016). In this study, we used BSP to detect the methylation of CpG at the *AKT2* locus, and differences were detected between *G. lucidum*-treated and control mice (Figure 5). Methylation of *AKT2* island 1 was 1.11% in AEG-APP/PS1, 3.33% in APP/PS1, and 2.22% in control mice (*p* < 0.05, Figure 5); while that of island 2 of *AKT2* was 2.31% for AEG-SAMP8, 4.31% for SAMP8, and 0.77% for control mice (*p* < 0.05, Figure 5).

p38α mitogen-activated protein kinase (MAPK) has been implicated in tau phosphorylation and inflammation; both of which are events associated with AD (Wang S. et al., 2014). The p38α MAPK pathway is activated by a dual phosphorylation at Thr180 and Tyr182 residues (Pinsetta et al., 2014), and structure- and ligand-based drug design of novel p38-alpha MAPK inhibitors may represent a realistic strategy in the fight against AD. The methylation of *MAPK3* detected in this study was 3.20% in AEG-APP/PS1, 0.80% in AEG-SAMP8, 0.80% in APP/PS1, 1.26% in SAMP8, and 2.00% in control mice. These data demonstrate that alcohol extracts of *G. lucidum* can regulate DNA methylation, and we hypothesize that *AKT2* and *MAPK3* are potential methylation targets in AD; however, further studies are required.

The importance of methylation in aging is linked with another epigenetic modification, histone acetylation (Zeng et al., 2015; Wang et al., 2018). A global reduction in gene expression, referred to as an epigenetic blockade, is observed in AD and may be regulated by post-translational modifications of histones (Gräff et al., 2012). Histone H3 is strongly regulated by phosphorylation and the H3 sites, S57 and T58, exhibit lower levels of phosphorylation in the 5XFAD model than those detected in wild type controls, implicating these sites in the epigenetic blockade associated with neurodegeneration pathology (Anderson et al., 2015). Epigenetic modifications, such as histone acetylation and deacetylation, are responsible for maintaining chromatin stability (Mahgoub and Monteggia, 2014). Treatment modalities using various HDAC inhibitors, which can enhance synaptic plasticity, learning and memory, have emerged as potential new strategies for therapeutic intervention in neurodegenerative diseases, including AD, Huntington’s disease, and Parkinson’s disease (Konsoula and Barile, 2012). Analysis of polymorphisms of *DNMT3A* and *DNMT3B* support a major role for these loci in the pathogenesis of late-onset AD and these polymorphisms can be used as stratification markers to predict an individual’s susceptibility to late-onset AD (Coppedè et al., 2012; Ling et al., 2016). The immunohistochemistry results reported here demonstrate that the Histone H3 DNA methyltransferases, DNMT3A and DNMT3B, were up-regulated in response to treatment with alcohol extracts from *G. lucidum* (Figure 7), indicating that *G. lucidum* can influence these DNA methyltransferases to regulate the DNA methylation, and that this may be an important signaling pathway influenced by *G. lucidum* in delaying the progress of AD and/or aging. Overall, we conclude that *G. lucidum* alcohol extracts, including ganoderic acid and lucidone A, are the main active extracts that can contribute to delaying AD progression.

## Ethics Statement

The animal protocols used in this work were approved by the Institutional Animal Care and Use Committee of the Center of Laboratory Animals of the Guangdong Institute of Microbiology.

## Author Contributions

All the authors participated in designing this study, carrying out the computational analyses, and writing the manuscript. YG and GL fed the mice, collected the physiological data and fecal samples and extracted microbial DNA. GL, YG, and XT helped to collect the mice physiological data and brain samples. GL, DC, YG, DM, OS, CX, and TY collected data regarding the microbial metabolic networks and proteomic analysis. XT and GL helped with the histopathological examination. DC, YG, DW, XT, and GL carried out the immunohistochemistry staining. DC, YX, YB, and QW helped to design the study and to develop the genomic analysis tools and reviewed the manuscript. All authors have read and approved the final manuscript.

## Conflict of Interest Statement

OS and YX are employed by Guangdong Yuewei Edible Fungi Technology Co., Ltd. The remaining authors declare that the research was conducted in the absence of any commercial or financial relationships that could be construed as a potential conflict of interest.

## Footnotes

1 http://deweylab.biostat.wisc.edu/rsem/

2 http://www.bioconductor.org/packages/2.12/bioc/html/edgeR.html

3 https://github.com/tanghaibao/Goatools

4 http://kobas.cbi.pku.edu.cn/

## References

[B1] Anderson K. W. Mast N. Pikuleva I. A. Turko I. V. (2015). Histone H3 Ser57 and Thr58 phosphorylation in the brain of 5XFAD mice. *FEBS Open Bio.* 5 550–556. 10.1016/j.fob.2015.06.009 26199864 PMC4506931

[B2] Cabili M. N. Trapnell C. Goff L. Koziol M. Tazon-Vega B. Regev A. (2011). Integrative annotation of human large intergenic noncoding RNAs reveals global properties and specific subclasses. *Genes Dev.* 25 1915–1927. 10.1101/gad.17446611 21890647 PMC3185964

[B3] Chen D. L. Zhang P. Lin L. Zhang H. M. Deng S. D. Wu Z. Q. (2014). Protective effects of bajijiasu in a rat model of Aβ25-35-induced neurotoxicity. *J. Ethnopharmacol.* 154 206–217. 10.1016/j.jep.2014.04.004 24742752

[B4] Chen D. L. Zheng C. Q. Yang J. Li J. Su J. Y. Xie Y. Z. (2017). Immunomodulatory activities of a fungal protein extracted from *Hericium erinaceus* through regulating the gut microbiota. *Front. Immunol.* 8:666. 10.3389/fimmu.2017.00666 28713364 PMC5492111

[B5] Chen S. Li X. Yong T. Wang Z. Su J. Jiao C. (2017). Cytotoxic lanostane-type triterpenoids from the fruiting bodies of *Ganoderma lucidum* and their structure-activity relationships. *Oncotarget* 8 10071–10084. 10.18632/oncotarget.14336 28052025 PMC5354642

[B6] Choi S. Nguyen V. T. Tae N. Lee S. Ryoo S. Min B. S. (2014). Anti-inflammatory and heme oxygenase-1 inducing activities of lanostane triterpenes isolated from mushroom *Ganoderma lucidum* in RAW264.7 cells. *Toxicol. Appl. Pharmacol.* 280 434–442. 10.1016/j.taap.2014.09.007 25239868

[B7] Cilerdžić J. Vukojević J. Stajić M. Stanojkovič T. Glamoèlija J. (2014). Biological activity of *Ganoderma lucidum* basidiocarps cultivated on alternative and commercial substrate. *J. Ethnopharmacol.* 155 312–319. 10.1016/j.jep.2014.05.036 24879959

[B8] Cohen N. Cohen J. Asatiani M. D. Varshney V. K. Yu H. T. Yang Y. C. (2014). Chemical composition and nutritional and medicinal value of fruit bodies and submerged cultured mycelia of culinary-medicinal higher *Basidiomycetes* mushrooms. *Int. J. Med. Mushrooms* 16 273–291. 10.1615/IntJMedMushr.v16.i3.80 24941169

[B9] Coppedè F. Zitarosa M. T. Migheli F. Lo Gerfo A. Bagnoli S. Dardano A. (2012). DNMT3B promoter polymorphisms and risk of late onset Alzheimer’s disease. *Curr. Alzheimer Res.* 9 550–554. 10.2174/156720512800618062 22272627

[B10] Coppieters N. Dieriks B. V. Lill C. Faull R. L. Curtis M. A. Dragunow M. (2014). Global changes in DNA methylation and hydroxymethylation in Alzheimer’s disease human brain. *Neurobiol. Aging* 35 1334–1344. 10.1016/j.neurobiolaging.2013.11.031 24387984

[B11] De Jager P. L. Srivastava G. Lunnon K. Burgess J. Schalkwyk L. C. Yu L. (2014). Alzheimer’s disease: early alterations in brain DNA methylation at ANK1 BIN1 RHBDF2 and other loci. *Nat. Neurosci.* 17 1156–1163. 10.1038/nn.3786 25129075 PMC4292795

[B12] Deepalakshmi K. Mirunalini S. Krishnaveni M. Arulmozhi V. (2013). In vitro and in vivo antioxidant potentials of an ethanolic extract of *Ganoderma lucidum* in rat mammary carcinogenesis. *Chin. J. Nat. Med*. 11 621–627. 10.1016/S1875-5364(13)60072-2 24345503

[B13] Gräff J. Rei D. Guan J. S. Wang W. Y. Seo J. Hennig K. M. (2012). An epigenetic blockade of cognitive functions in the neurodegenerating brain. *Nature* 483 222–226. 10.1038/nature10849 22388814 PMC3498952

[B14] Harrison I. F. Dexter D. T. (2013). Epigenetic targeting of histone deacetylase: therapeutic potential in Parkinson’s disease? *Pharmacol. Ther*. 140 34–52. 10.1016/j.pharmthera.2013.05.010 23711791

[B15] Konsoula Z. Barile F. A. (2012). Epigenetic histone acetylation and deacetylation mechanisms in experimental models of neurodegenerative disorders. *J. Pharmacol. Toxicol. Methods* 66 215–220. 10.1016/j.vascn.2012.08.001 22902970

[B16] Krzywinski M. Schein J. Birol I. Connors J. Gascoyne R. Horsman D. (2009). Circos: an information aesthetic for comparative genomics. *Genome Res.* 19 1639–1645. 10.1101/gr.092759.109 19541911 PMC2752132

[B17] Lee Y. H. Kim J. H. Song C. H. Jang K. J. Kim C. H. Kang J. S. (2016). Ethanol extract of *Ganoderma lucidum* augments cellular anti-oxidant defense through activation of Nrf2/HO-1. *J. Pharmacopuncture* 19 59–69. 10.3831/KPI.2016.19.008 27280051 PMC4887753

[B18] Liang C. Y. Liang Y. M. Liu H. Z. Zhu D. M. Hou S. Z. Wu Y. Y. (2017). Effect of *Dendrobium officinale* on D-galactose-induced aging mice. *Chin. J. Integr. Med.* 10.1007/s11655-016-2631-x [Epub ahead of print]. 28083812

[B19] Ling C. Fangyu D. Wanhua H. Kelong C. Zhimin W. Yuting Z. (2016). DNMT3A rs1550117 polymorphism is associated with late-onset Alzheimer’s disease in a Chinese population. *Am. J. Alzheimers Dis. Other Demen.* 31 278–281. 10.1177/1533317515603688 26371346 PMC10852886

[B20] Ma F. F. Cao D. D. Ouyang S. Tang R. Liu Z. Li Y. (2016). Hypermethylation of AKT2 gene is associated with neural-tube defects in fetus. *Placenta* 48 80–86. 10.1016/j.placenta.2016.10.010 27871477

[B21] Mahgoub M. Monteggia L. M. (2014). A role for histone deacetylases in the cellular and behavioral mechanisms underlying learning and memory. *Learn. Mem.* 21 564–568. 10.1101/lm.036012.114 25227251 PMC4175496

[B22] Pinsetta F. R. Taft C. A. de Paula da Silva C. H. (2014). Structure- and ligand-based drug design of novel p38-alpha MAPK inhibitors in the fight against the Alzheimer’s disease. *J. Biomol. Struct. Dyn.* 32 1047–1063. 10.1080/07391102.2013.803441 23805842

[B23] Ren J. Yang L. Zhu L. Xu X. Ceylan A. F. Guo W. (2017). Akt2 ablation prolongs life span and improves myocardial contractile function with adaptive cardiac remodeling: role of Sirt1-mediated autophagy regulation. *Aging Cell.* 16 976–987. 10.1111/acel.12616 28681509 PMC5595687

[B24] Sanchez-Mut J. V. Aso E. Panayotis N. Lott I. Dierssen M. Rabano A. (2013). DNA methylation map of mouse and human brain identifies target genes in Alzheimer’s disease. *Brain* 136(Pt 10), 3018–3027. 10.1093/brain/awt237 24030951 PMC3784285

[B25] Trapnell C. Hendrickson D. G. Sauvageau M. Goff L. Rinn J. L. Pachter L. (2013). Differential analysis of gene regulation at transcript resolution with RNA-seq. *Nat. Biotechnol.* 31 46–53. 10.1038/nbt.2450 23222703 PMC3869392

[B26] Wang D. B. Kinoshita C. Kinoshita Y. Sopher B. L. Uo T. Lee R. J. (2018). Neuronal susceptibility to beta-Amyloid toxicity and ischemic injury involves histone deacetylase-2 regulation of endophilin-B1. *Brain Pathol.* 29 164–175. 10.1111/bpa.12647 30028551 PMC6339848

[B27] Wang J. Cao B. Zhao H. Feng J. (2017). Emerging roles of ganoderma lucidum in anti-aging. *Aging Dis.* 8 691–707. 10.14336/AD.2017.0410 29344411 PMC5758346

[B28] Wang S. Zhang C. Sheng X. Zhang X. Wang B. Zhang G. (2014). Peripheral expression of MAPK pathways in Alzheimer’s and Parkinson’s diseases. *J. Clin. Neurosci.* 21 810–814. 10.1016/j.jocn.2013.08.017 24405770

[B29] Wang X. Li G. J. Hu H. X. Ma C. Ma D. H. Liu X. L. (2016). Cerebral mTOR signal and pro-inflammatory cytokines in Alzheimer’s disease rats. *Transl. Neurosci.* 7 151–157. 10.1515/tnsci-2016-0022 28123835 PMC5234524

[B30] Wang X. M. Zhang J. Wu L. H. Zhao Y. L. Li T. Li J. Q. (2014). A mini-review of chemical composition and nutritional value of edible wild-grown mushroom from China. *Food Chem.* 151 279–285. 10.1016/j.foodchem.2013.11.062 24423533

[B31] Wang Y. Xiang L. Matsuura A. Zhang Y. Huang Q. Qi J. (2010). Ganodermasides A and, B., two novel anti-aging ergosterols from spores of a medicinal mushroom *Ganoderma lucidum* on yeast via UTH1 gene. *Bioorg. Med. Chem*. 18 999–1002. 10.1016/j.bmc.2009.12.070 20093034

[B32] Weng Y. Lu J. Xiang L. Matsuura A. Zhang Y. Huang Q. (2011). Ganodermasides C and, D., two new anti-aging ergosterols from spores of the medicinal mushroom *Ganoderma lucidum*. *Biosci. Biotechnol. Biochem.* 75 800–803. 10.1271/bbb.100918 21512225

[B33] Zeng G. F. Zhang Z. Y. Lu L. Xiao D. Q. Zong S. H. He J. M. (2013). Protective effects of ginger root extract on Alzheimer disease-induced behavioral dysfunction in rats. *Rejuvenation Res.* 16 124–133. 10.1089/rej.2012.1389 23374025

[B34] Zeng H. Huang P. Wang X. Wu J. Wu M. Huang J. (2015). Galangin-induced down-regulation of BACE1 by epigenetic mechanisms in SH-SY5Y cells. *Neuroscience* 294 172–181. 10.1016/j.neuroscience.2015.02.054 25779965

[B35] Zhong L. Huang F. Shi H. Wu H. Zhang B. Wu X. (2016). Qing’E formula alleviates the aging process in D-galactose-induced aging mice. *Biomed. Rep.* 5 101–106. 10.3892/br.2016.667 27347412 PMC4906976

[B36] Zusso M. Barbierato M. Facci L. Skaper S. D. Giusti P. (2018). Neuroepigenetics and alzheimer’s disease: an update. *J. Alzheimers Dis.* 64 671–688. 10.3233/JAD-180259 29991138

