# Supplementary material for: Alcohol Extracts From Ganoderma lucidum Delay the Progress of Alzheimer’s Disease by Regulating DNA Methylation in Rodents
Source: Front Pharmacol. 2019 Mar 26;10:272. doi: 10.3389/fphar.2019.00272 (PMC6444160; doi:10.3389/fphar.2019.00272)
Supplement: Supplementary file 1 [file Table_1.DOC]

# Supplementary materials

## Table S1

Table S1 KEGG pathway analysis Control vs D-galactose- induced deficient rats

| Pathway_ID | Pathway | DEG_number | Total_number | *p*-value | FDR |
| --- | --- | --- | --- | --- | --- |
| ko04972 | Pancreatic secretion | 19 | 96 | 0.00045 | 0.0585 |
| ko04080 | Neuroactive ligand-receptor interaction | 43 | 298 | 0.00051 | 0.0585 |
| ko00363 | Bisphenol degradation | 3 | 3 | 0.00063 | 0.0585 |
| ko04724 | Glutamatergic synapse | 20 | 110 | 0.00101 | 0.0701 |
| ko04924 | Renin secretion | 13 | 62 | 0.00202 | 0.1119 |
| ko04725 | Cholinergic synapse | 18 | 107 | 0.00426 | 0.1965 |
| ko04974 | Protein digestion and absorption | 15 | 87 | 0.00687 | 0.2404 |
| ko00261 | Monobactam biosynthesis | 2 | 2 | 0.00738 | 0.2404 |
| ko04973 | Carbohydrate digestion and absorption | 8 | 37 | 0.01180 | 0.2404 |
| ko04922 | Glucagon signaling pathway | 14 | 84 | 0.01185 | 0.2404 |
| ko00480 | Glutathione metabolism | 10 | 52 | 0.01195 | 0.2404 |
| ko00980 | Metabolism of xenobiotics by cytochrome P450 | 10 | 52 | 0.01195 | 0.2404 |
| ko04723 | Retrograde endocannabinoid signaling | 16 | 101 | 0.01203 | 0.2404 |
| ko04360 | Axon guidance | 24 | 172 | 0.01215 | 0.2404 |
| ko00982 | Drug metabolism - cytochrome P450 | 10 | 53 | 0.01363 | 0.2518 |
| ko04261 | Adrenergic signaling in cardiomyocytes | 20 | 140 | 0.01642 | 0.2700 |
| ko04666 | Fc gamma R-mediated phagocytosis | 13 | 79 | 0.01657 | 0.2700 |
| ko00920 | Sulfur metabolism | 4 | 13 | 0.02066 | 0.3179 |
| ko00532 | Glycosaminoglycan biosynthesis-chondroitin sulfate/dermatan sulfate | 5 | 20 | 0.02420 | 0.3325 |
| ko04015 | Rap1 signaling pathway | 26 | 202 | 0.02436 | 0.3325 |
| ko03320 | PPAR signaling pathway | 12 | 75 | 0.02551 | 0.3325 |
| ko00561 | Glycerolipid metabolism | 10 | 59 | 0.02761 | 0.3325 |
| ko01212 | Fatty acid metabolism | 9 | 51 | 0.02817 | 0.3325 |
| ko04151 | PI3K-Akt signaling pathway | 37 | 313 | 0.02896 | 0.3325 |
| ko04610 | Complement and coagulation cascades | 12 | 77 | 0.03069 | 0.3325 |
| ko04022 | cGMP-PKG signaling pathway | 21 | 159 | 0.03135 | 0.3325 |
| ko04662 | B cell receptor signaling pathway | 11 | 69 | 0.03241 | 0.3325 |
| ko04214 | Apoptosis - fly | 10 | 62 | 0.03752 | 0.3712 |
| ko04961 | Endocrine and other factor-regulated calcium reabsorption | 8 | 46 | 0.04038 | 0.3758 |
| ko04975 | Fat digestion and absorption | 7 | 38 | 0.04070 | 0.3758 |
| ko00120 | Primary bile acid biosynthesis | 4 | 16 | 0.04280 | 0.3824 |
| ko04911 | Insulin secretion | 12 | 82 | 0.04686 | 0.4056 |

## Table S2

Table S2 GO analysis Control vs D-galactose- induced deficient rats

| GO_Term | Cluster frequency | Genome frequency of use | Corrected *p*-value |
| --- | --- | --- | --- |
| GO:0044459 plasma membrane part | 235 out of 1639 genes, 14.3% | 1860 out of 20039 genes, 9.3% | 1.63E-09 |
| GO:0031226 intrinsic component of plasma membrane | 129 out of 1639 genes, 7.9% | 937 out of 20039 genes, 4.7% | 1.31E-06 |
| GO:0005887 integral component of plasma membrane | 121 out of 1639 genes, 7.4% | 886 out of 20039 genes, 4.4% | 6.79E-06 |
| GO:0031982 vesicle | 297 out of 1639 genes, 18.1% | 2801 out of 20039 genes, 14.0% | 0.00034 |
| GO:0044707 single-multicellular organism process | 434 out of 1639 genes, 26.5% | 4282 out of 20039 genes, 21.4% | 0.00065 |
| GO:0015075 ion transmembrane transporter activity | 101 out of 1639 genes, 6.2% | 772 out of 20039 genes, 3.9% | 0.00123 |
| GO:0022857 transmembrane transporter activity | 116 out of 1639 genes, 7.1% | 923 out of 20039 genes, 4.6% | 0.00155 |
| GO:0006811 ion transport | 129 out of 1639 genes, 7.9% | 1025 out of 20039 genes, 5.1% | 0.0019 |
| GO:0022890 inorganic cation transmembrane transporter activity | 68 out of 1639 genes, 4.1% | 468 out of 20039 genes, 2.3% | 0.00196 |
| GO:0098660 inorganic ion transmembrane transport | 70 out of 1639 genes, 4.3% | 466 out of 20039 genes, 2.3% | 0.00207 |
| GO:0022892 substrate-specific transporter activity | 122 out of 1639 genes, 7.4% | 997 out of 20039 genes, 5.0% | 0.00319 |
| GO:0098590 plasma membrane region | 90 out of 1639 genes, 5.5% | 689 out of 20039 genes, 3.4% | 0.00338 |
| GO:0022891 substrate-specific transmembrane transporter activity | 106 out of 1639 genes, 6.5% | 842 out of 20039 genes, 4.2% | 0.00402 |
| GO:0046873 metal ion transmembrane transporter activity | 55 out of 1639 genes, 3.4% | 361 out of 20039 genes, 1.8% | 0.00435 |
| GO:0005216 ion channel activity | 57 out of 1639 genes, 3.5% | 379 out of 20039 genes, 1.9% | 0.00441 |
| GO:0008324 cation transmembrane transporter activity | 77 out of 1639 genes, 4.7% | 564 out of 20039 genes, 2.8% | 0.0047 |
| GO:0055085 transmembrane transport | 116 out of 1639 genes, 7.1% | 914 out of 20039 genes, 4.6% | 0.00488 |
| GO:0022838 substrate-specific channel activity | 58 out of 1639 genes, 3.5% | 390 out of 20039 genes, 1.9% | 0.0052 |
| GO:0005085 guanyl-nucleotide exchange factor activity | 32 out of 1639 genes, 2.0% | 170 out of 20039 genes, 0.8% | 0.00576 |
| GO:0034220 ion transmembrane transport | 87 out of 1639 genes, 5.3% | 637 out of 20039 genes, 3.2% | 0.00591 |
| GO:0048731 system development | 346 out of 1639 genes, 21.1% | 3385 out of 20039 genes, 16.9% | 0.00887 |
| GO:0042995 cell projection | 167 out of 1639 genes, 10.2% | 1495 out of 20039 genes, 7.5% | 0.00911 |
| GO:0065008 regulation of biological quality | 288 out of 1639 genes, 17.6% | 2743 out of 20039 genes, 13.7% | 0.00928 |
| GO:0098662 inorganic cation transmembrane transport | 61 out of 1639 genes, 3.7% | 404 out of 20039 genes, 2.0% | 0.00937 |
| GO:0006812 cation transport | 91 out of 1639 genes, 5.6% | 684 out of 20039 genes, 3.4% | 0.01041 |
| GO:0098589 membrane region | 104 out of 1639 genes, 6.3% | 848 out of 20039 genes, 4.2% | 0.01049 |
| GO:0048513 animal organ development | 267 out of 1639 genes, 16.3% | 2518 out of 20039 genes, 12.6% | 0.01094 |
| GO:0001594 trace-amine receptor activity | 8 out of 1639 genes, 0.5% | 16 out of 20039 genes, 0.1% | 0.01152 |
| GO:0015077 monovalent inorganic cation transmembrane transporter activity | 50 out of 1639 genes, 3.1% | 330 out of 20039 genes, 1.6% | 0.01334 |
| GO:0030001 metal ion transport | 68 out of 1639 genes, 4.1% | 472 out of 20039 genes, 2.4% | 0.01353 |
| GO:0022803 passive transmembrane transporter activity | 60 out of 1639 genes, 3.7% | 421 out of 20039 genes, 2.1% | 0.01377 |
| GO:0015267 channel activity | 60 out of 1639 genes, 3.7% | 421 out of 20039 genes, 2.1% | 0.01377 |
| GO:0010817 regulation of hormone levels | 59 out of 1639 genes, 3.6% | 391 out of 20039 genes, 2.0% | 0.01396 |
| GO:0005215 transporter activity | 137 out of 1639 genes, 8.4% | 1184 out of 20039 genes, 5.9% | 0.01483 |

## Table S3

Table S3 KEGG pathway analysis of AEG-treated vs D-galactose- induced deficient rats

| Pathway_ID | Pathway | DEG_number | Total_number | *p*-value | FDR |
| --- | --- | --- | --- | --- | --- |
| ko04915 | Estrogen signaling pathway | 5 | 93 | 0.00121 | 0.11097 |
| ko00140 | Steroid hormone biosynthesis | 4 | 57 | 0.00143 | 0.11097 |
| ko04730 | Long-term depression | 4 | 59 | 0.00163 | 0.11097 |
| ko04924 | Renin secretion | 4 | 62 | 0.00196 | 0.11097 |
| ko04022 | cGMP-PKG signaling pathway | 6 | 159 | 0.00246 | 0.11097 |
| ko04724 | Glutamatergic synapse | 5 | 110 | 0.00255 | 0.11097 |
| ko04662 | B cell receptor signaling pathway | 4 | 69 | 0.00289 | 0.11097 |
| ko04726 | Serotonergic synapse | 5 | 116 | 0.00320 | 0.11097 |
| ko04917 | Prolactin signaling pathway | 4 | 74 | 0.00373 | 0.11475 |
| ko04911 | Insulin secretion | 4 | 82 | 0.00538 | 0.14865 |
| ko04540 | Gap junction | 4 | 85 | 0.00611 | 0.14865 |
| ko04912 | GnRH signaling pathway | 4 | 87 | 0.00663 | 0.14865 |
| ko04072 | Phospholipase D signaling pathway | 5 | 140 | 0.00709 | 0.14865 |
| ko00623 | Toluene degradation | 1 | 1 | 0.00859 | 0.14865 |
| ko00364 | Fluorobenzoate degradation | 1 | 1 | 0.00859 | 0.14865 |
| ko00361 | Chlorocyclohexane and chlorobenzene degradation | 1 | 1 | 0.00859 | 0.14865 |
| ko04725 | Cholinergic synapse | 4 | 107 | 0.01349 | 0.20464 |
| ko04668 | TNF signaling pathway | 4 | 108 | 0.01392 | 0.20464 |
| ko00830 | Retinol metabolism | 3 | 60 | 0.01478 | 0.20464 |
| ko04720 | Long-term potentiation | 3 | 60 | 0.01478 | 0.20464 |
| ko00230 | Purine metabolism | 5 | 170 | 0.01551 | 0.20464 |
| ko04918 | Thyroid hormone synthesis | 3 | 63 | 0.01684 | 0.20593 |
| ko00261 | Monobactam biosynthesis | 1 | 2 | 0.01710 | 0.20593 |
| ko04071 | Sphingolipid signaling pathway | 4 | 120 | 0.01975 | 0.22789 |
| ko00363 | Bisphenol degradation | 1 | 3 | 0.02554 | 0.28298 |
| ko04015 | Rap1 signaling pathway | 5 | 202 | 0.02999 | 0.31950 |
| ko00730 | Thiamine metabolism | 1 | 4 | 0.03391 | 0.34535 |
| ko04922 | Glucagon signaling pathway | 3 | 84 | 0.03553 | 0.34535 |
| ko00591 | Linoleic acid metabolism | 2 | 35 | 0.03616 | 0.34535 |
| ko04211 | Longevity regulating pathway | 3 | 89 | 0.04108 | 0.35458 |
| ko04921 | Oxytocin signaling pathway | 4 | 152 | 0.04183 | 0.35458 |
| ko04916 | Melanogenesis | 3 | 90 | 0.04224 | 0.35458 |
| ko04713 | Circadian entrainment | 3 | 90 | 0.04224 | 0.35458 |
| ko04960 | Aldosterone-regulated sodium reabsorption | 2 | 41 | 0.04821 | 0.38119 |
| ko04066 | HIF-1 signaling pathway | 3 | 96 | 0.04954 | 0.38119 |
| ko04972 | Pancreatic secretion | 3 | 96 | 0.04954 | 0.38119 |

## Table S4

Table S4 GO analysis of AEG-treated and SAMP8 mice

| **GO_Term** | **Cluster frequency** | **Genome frequency of use** | **Corrected *p*-value** |
| --- | --- | --- | --- |
| GO:0005515 protein binding | 1812 out of 2925 genes, 61.9% | 10532 out of 20795 genes, 50.6% | 3.67E-37 |
| GO:0005737 cytoplasm | 1609 out of 2925 genes, 55.0% | 9536 out of 20795 genes, 45.9% | 5.37E-24 |
| GO:0005488 binding | 2200 out of 2925 genes, 75.2% | 14125 out of 20795 genes, 67.9% | 1.18E-17 |
| GO:0043226 organelle | 1891 out of 2925 genes, 64.6% | 11933 out of 20795 genes, 57.4% | 2.76E-15 |
| GO:0048518 positive regulation of biological process | 895 out of 2925 genes, 30.6% | 4984 out of 20795 genes, 24.0% | 3.01E-15 |
| GO:0044424 intracellular part | 1948 out of 2925 genes, 66.6% | 12372 out of 20795 genes, 59.5% | 7.61E-15 |
| GO:0005622 intracellular | 2016 out of 2925 genes, 68.9% | 12877 out of 20795 genes, 61.9% | 8.61E-15 |
| GO:0042995 cell projection | 362 out of 2925 genes, 12.4% | 1699 out of 20795 genes, 8.2% | 1.13E-14 |
| GO:0005856 cytoskeleton | 376 out of 2925 genes, 12.9% | 1819 out of 20795 genes, 8.7% | 3.63E-13 |
| GO:0051128 regulation of cellular component organization | 438 out of 2925 genes, 15.0% | 2178 out of 20795 genes, 10.5% | 9.26E-13 |
| GO:0097458 neuron part | 264 out of 2925 genes, 9.0% | 1187 out of 20795 genes, 5.7% | 2.40E-12 |
| GO:0016043 cellular component organization | 794 out of 2925 genes, 27.1% | 4442 out of 20795 genes, 21.4% | 3.31E-12 |
| GO:0048522 positive regulation of cellular process | 779 out of 2925 genes, 26.6% | 4370 out of 20795 genes, 21.0% | 1.45E-11 |
| GO:0044444 cytoplasmic part | 1099 out of 2925 genes, 37.6% | 6544 out of 20795 genes, 31.5% | 1.76E-11 |
| GO:0098805 whole membrane | 358 out of 2925 genes, 12.2% | 1760 out of 20795 genes, 8.5% | 2.31E-11 |
| GO:0044763 single-organism cellular process | 1770 out of 2925 genes, 60.5% | 11210 out of 20795 genes, 53.9% | 2.92E-11 |
| GO:0071840 cellular component organization or biogenesis | 813 out of 2925 genes, 27.8% | 4606 out of 20795 genes, 22.1% | 3.15E-11 |
| GO:0032879 regulation of localization | 436 out of 2925 genes, 14.9% | 2226 out of 20795 genes, 10.7% | 1.04E-10 |
| GO:0051179 localization | 730 out of 2925 genes, 25.0% | 4091 out of 20795 genes, 19.7% | 1.40E-10 |
| GO:0043005 neuron projection | 208 out of 2925 genes, 7.1% | 909 out of 20795 genes, 4.4% | 1.44E-10 |
| GO:0044430 cytoskeletal part | 288 out of 2925 genes, 9.8% | 1368 out of 20795 genes, 6.6% | 1.63E-10 |
| GO:0009987 cellular process | 2116 out of 2925 genes, 72.3% | 13816 out of 20795 genes, 66.4% | 5.12E-10 |
| GO:0010646 regulation of cell communication | 502 out of 2925 genes, 17.2% | 2663 out of 20795 genes, 12.8% | 7.65E-10 |
| GO:0043229 intracellular organelle | 1699 out of 2925 genes, 58.1% | 10830 out of 20795 genes, 52.1% | 9.64E-10 |

## Table S5

Table S5 KEGG pathway analysis of AEG-treated and SAMP8 mice

| **Pathway_ID** | **Pathway** | **DEG_number** | **Total_number** | ***p*-value** | **FDR** |
| --- | --- | --- | --- | --- | --- |
| ko04728 | Dopaminergic synapse | 42 | 128 | 9.12E-07 | 0.000255241 |
| ko04919 | Thyroid hormone signaling pathway | 34 | 112 | 5.97E-05 | 0.008362795 |
| ko04724 | Glutamatergic synapse | 33 | 111 | 0.000119 | 0.008715695 |
| ko04920 | Adipocytokine signaling pathway | 24 | 72 | 0.000144 | 0.008715695 |
| ko04713 | Circadian entrainment | 29 | 95 | 0.000182 | 0.008715695 |
| ko04921 | Oxytocin signaling pathway | 42 | 156 | 0.000187 | 0.008715695 |
| ko04726 | Serotonergic synapse | 36 | 129 | 0.000245 | 0.009415196 |
| ko04721 | Synaptic vesicle cycle | 21 | 62 | 0.000287 | 0.009415196 |
| ko04360 | Axon guidance | 45 | 174 | 0.000303 | 0.009415196 |
| ko04070 | Phosphatidylinositol signaling system | 28 | 94 | 0.000366 | 0.010257145 |
| ko04972 | Pancreatic secretion | 29 | 100 | 0.000476 | 0.01212008 |
| ko04520 | Adherens junction | 22 | 71 | 0.00084 | 0.018139696 |
| ko04725 | Cholinergic synapse | 30 | 108 | 0.000845 | 0.018139696 |
| ko04540 | Gap junction | 25 | 85 | 0.000907 | 0.018139696 |
| ko04530 | Tight junction | 36 | 138 | 0.000978 | 0.018250107 |
| ko04062 | Chemokine signaling pathway | 46 | 189 | 0.00108 | 0.01890294 |
| ko04261 | Adrenergic signaling in cardiomyocytes | 37 | 145 | 0.001297 | 0.021361875 |
| ko04912 | GnRH signaling pathway | 25 | 88 | 0.001564 | 0.024329262 |
| ko04723 | Retrograde endocannabinoid signaling | 27 | 99 | 0.002007 | 0.0289666 |
| ko04925 | Aldosterone synthesis and secretion | 24 | 85 | 0.002104 | 0.0289666 |
| ko04611 | Platelet activation | 31 | 119 | 0.002172 | 0.0289666 |
| ko04971 | Gastric acid secretion | 21 | 72 | 0.002527 | 0.032163511 |
| ko04320 | Dorso-ventral axis formation | 10 | 25 | 0.002848 | 0.034674299 |
| ko04727 | GABAergic synapse | 23 | 83 | 0.003347 | 0.038580295 |
| ko00564 | Glycerophospholipid metabolism | 25 | 93 | 0.00356 | 0.038580295 |
| ko04961 | Endocrine and other factor-regulated calcium reabsorption | 16 | 51 | 0.003582 | 0.038580295 |
| ko04922 | Glucagon signaling pathway | 26 | 99 | 0.004213 | 0.043695058 |
| ko04391 | Hippo signaling pathway - fly | 19 | 66 | 0.004643 | 0.04643124 |
| ko04071 | Sphingolipid signaling pathway | 30 | 120 | 0.004888 | 0.047193102 |

## Table S6

Table S6 GO analysis of AEG-treated and APP/PS1 mice

| **GO_Term** | **Cluster frequency** | **Genome frequency of use** | **Corrected *p*-value** |
| --- | --- | --- | --- |
| GO:0005515 protein binding | 1779 out of 2993 genes, 59.4% | 10532 out of 20795 genes, 50.6% | 1.42E-22 |
| GO:0005737 cytoplasm | 1601 out of 2993 genes, 53.5% | 9536 out of 20795 genes, 45.9% | 7.31E-17 |
| GO:0043226 organelle | 1904 out of 2993 genes, 63.6% | 11933 out of 20795 genes, 57.4% | 3.02E-11 |
| GO:0016043 cellular component organization | 803 out of 2993 genes, 26.8% | 4442 out of 20795 genes, 21.4% | 4.51E-11 |
| GO:0044424 intracellular part | 1963 out of 2993 genes, 65.6% | 12372 out of 20795 genes, 59.5% | 6.63E-11 |
| GO:0005622 intracellular | 2032 out of 2993 genes, 67.9% | 12877 out of 20795 genes, 61.9% | 1.01E-10 |
| GO:0071840 cellular component organization or biogenesis | 826 out of 2993 genes, 27.6% | 4606 out of 20795 genes, 22.1% | 1.13E-10 |
| GO:0005488 binding | 2203 out of 2993 genes, 73.6% | 14125 out of 20795 genes, 67.9% | 2.12E-10 |
| GO:0005856 cytoskeleton | 369 out of 2993 genes, 12.3% | 1819 out of 20795 genes, 8.7% | 3.62E-10 |
| GO:0051179 localization | 740 out of 2993 genes, 24.7% | 4091 out of 20795 genes, 19.7% | 7.88E-10 |
| GO:0010646 regulation of cell communication | 510 out of 2993 genes, 17.0% | 2663 out of 20795 genes, 12.8% | 1.64E-09 |
| GO:0048583 regulation of response to stimulus | 584 out of 2993 genes, 19.5% | 3127 out of 20795 genes, 15.0% | 2.44E-09 |
| GO:0023051 regulation of signaling | 498 out of 2993 genes, 16.6% | 2620 out of 20795 genes, 12.6% | 1.22E-08 |
| GO:0044444 cytoplasmic part | 1099 out of 2993 genes, 36.7% | 6544 out of 20795 genes, 31.5% | 1.75E-08 |
| GO:0098772 molecular function regulator | 239 out of 2993 genes, 8.0% | 1111 out of 20795 genes, 5.3% | 3.65E-08 |
| GO:0048518 positive regulation of biological process | 865 out of 2993 genes, 28.9% | 4984 out of 20795 genes, 24.0% | 6.62E-08 |
| GO:0044707 single-multicellular organism process | 838 out of 2993 genes, 28.0% | 4823 out of 20795 genes, 23.2% | 1.32E-07 |
| GO:0043229 intracellular organelle | 1717 out of 2993 genes, 57.4% | 10830 out of 20795 genes, 52.1% | 1.63E-07 |
| GO:0098805 whole membrane | 343 out of 2993 genes, 11.5% | 1760 out of 20795 genes, 8.5% | 4.39E-07 |
| GO:0065009 regulation of molecular function | 439 out of 2993 genes, 14.7% | 2319 out of 20795 genes, 11.2% | 6.15E-07 |
| GO:0051128 regulation of cellular component organization | 416 out of 2993 genes, 13.9% | 2178 out of 20795 genes, 10.5% | 6.17E-07 |
| GO:0048522 positive regulation of cellular process | 763 out of 2993 genes, 25.5% | 4370 out of 20795 genes, 21.0% | 6.46E-07 |
| GO:0009966 regulation of signal transduction | 438 out of 2993 genes, 14.6% | 2316 out of 20795 genes, 11.1% | 7.49E-07 |
| GO:0043168 anion binding | 452 out of 2993 genes, 15.1% | 2430 out of 20795 genes, 11.7% | 8.01E-07 |

## Table S7

Table S7 KEGG pathway analysis of AEG-treated group vs APP/PS1 mice

| **Pathway_ID** | **Pathway** | **DEG_number** | **Total_number** | ***p*-value** | **FDR** |
| --- | --- | --- | --- | --- | --- |
| ko04724 | Glutamatergic synapse | 38 | 111 | 9.58E-07 | 0.000268175 |
| ko04010 | MAPK signaling pathway | 64 | 250 | 2.73E-05 | 0.003828103 |
| ko04972 | Pancreatic secretion | 31 | 100 | 8.34E-05 | 0.007786617 |
| ko00052 | Galactose metabolism | 13 | 32 | 0.00059 | 0.041273239 |
| ko04014 | Ras signaling pathway | 53 | 226 | 0.001239 | 0.045480243 |
| ko04512 | ECM-receptor interaction | 24 | 82 | 0.001261 | 0.045480243 |
| ko04974 | Protein digestion and absorption | 25 | 87 | 0.001347 | 0.045480243 |
| ko04730 | Long-term depression | 19 | 60 | 0.001422 | 0.045480243 |
| ko04919 | Thyroid hormone signaling pathway | 30 | 112 | 0.001642 | 0.045480243 |
| ko00521 | Streptomycin biosynthesis | 6 | 10 | 0.001714 | 0.045480243 |
| ko04728 | Dopaminergic synapse | 33 | 128 | 0.001982 | 0.045480243 |
| ko04922 | Glucagon signaling pathway | 27 | 99 | 0.002065 | 0.045480243 |
| ko04713 | Circadian entrainment | 26 | 95 | 0.002345 | 0.045480243 |
| ko04261 | Adrenergic signaling in cardiomyocytes | 36 | 145 | 0.002573 | 0.045480243 |
| ko04920 | Adipocytokine signaling pathway | 21 | 72 | 0.002588 | 0.045480243 |
| ko00524 | Butirosin and neomycin biosynthesis | 4 | 5 | 0.002599 | 0.045480243 |
| ko04062 | Chemokine signaling pathway | 44 | 189 | 0.003544 | 0.053833964 |
| ko04912 | GnRH signaling pathway | 24 | 88 | 0.003554 | 0.053833964 |
| ko04961 | Endocrine and other factor-regulated calcium reabsorption | 16 | 51 | 0.003653 | 0.053833964 |
| ko04020 | Calcium signaling pathway | 42 | 180 | 0.004131 | 0.05528411 |
| ko04015 | Rap1 signaling pathway | 48 | 212 | 0.004286 | 0.05528411 |
| ko04726 | Serotonergic synapse | 32 | 129 | 0.004372 | 0.05528411 |
| ko04151 | PI3K-Akt signaling pathway | 71 | 338 | 0.004688 | 0.05528411 |
| ko04925 | Aldosterone synthesis and secretion | 23 | 85 | 0.004739 | 0.05528411 |
